# Azobenzene-Based Amino Acids for the Photocontrol of Coiled-Coil Peptides

**DOI:** 10.1021/acs.bioconjchem.2c00534

**Published:** 2023-01-27

**Authors:** Niek S. A. Crone, Niek van Hilten, Alex van der Ham, Herre Jelger Risselada, Alexander Kros, Aimee L. Boyle

**Affiliations:** Leiden Institute of Chemistry, Leiden University, Einsteinweg 55, 2333 CCLeiden, The Netherlands

## Abstract

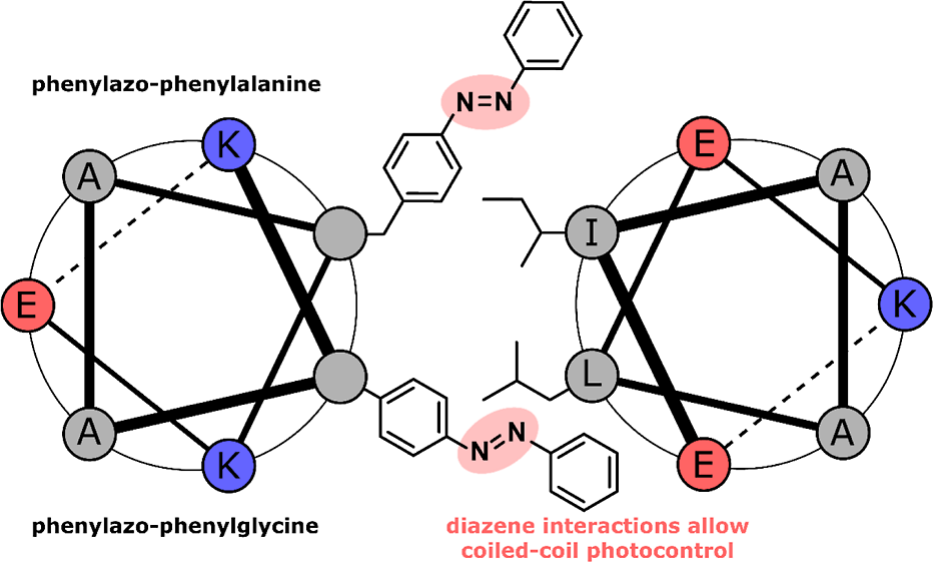

Coiled-coil peptides are high-affinity, selective, self-assembling binding motifs, making them attractive components for the preparation of functional biomaterials. Photocontrol of coiled-coil self-assembly allows for the precise localization of their activity. To rationally explore photoactivity in a model coiled coil, three azobenzene-containing amino acids were prepared and substituted into the hydrophobic core of the E_3_/K_3_ coiled-coil heterodimer. Two of the non-natural amino acids, **APhe1** and **APhe2**, are based on phenylalanine and differ in the presence of a carboxylic acid group. These have previously been demonstrated to modulate protein activity. When incorporated into peptide K_3_, coiled-coil binding strength was affected upon isomerization, with the two variants differing in their most folded state. The third azobenzene-containing amino acid, **APgly**, is based on phenylglycine and was prepared to investigate the effect of amino acid size on photoisomerization. When **APgly** is incorporated into the coiled coil, a 4.7-fold decrease in folding constant is observed upon trans-to-cis isomerization—the largest difference for all three amino acids. Omitting the methylene group between azobenzene and α-carbon was theorized to both position the diazene of **APgly** closer to the hydrophobic amino acids and reduce the possible rotations of the amino acid, with molecular dynamics simulations supporting these hypotheses. These results demonstrate the ability of photoswitchable amino acids to control coiled-coil assembly through disruption of the hydrophobic interface, a strategy that should be widely applicable.

## Introduction

Active control of coiled-coil peptide formation allows spatiotemporal control of their binding interactions, a property which has proven useful in synthetic materials and biological applications.^1−3^ In particular, the use of light to reversibly control peptide structures has been investigated because of the high resolution and biocompatibility of this strategy.^4−7^ Coiled-coil peptides can be designed to self-assemble with high affinity and selectivity,^8^ leading to them being widely employed for the de novo design of synthetic biomaterials.^9−11^ Previous efforts to reversibly photocontrol coiled-coil peptides relied on intramolecular cyclization to control peptide structures^12^ or the intermolecular connection of two separate coiled-coil strands with a photoswitchable linker.^1^ The incorporation of photoswitches into the binding interface of coiled coils has not yet been attempted and would provide a more direct route for control over their activity.

Single amino acid modifications in the hydrophobic core of coiled-coil forming peptides have been investigated, both to aid understanding of coiled-coil assembly and to introduce selectivity or functionality. The group of Hodges investigated substitution at the “a” position of a cysteine-cross-linked homotrimeric system to determine the effect on binding strength,^13^ and, in a separate study, the role of the “d” position in a cross-linked homodimeric peptide.^14^ Hydrophobic amino acids were found to be stabilizing at these positions, while polar amino acids were destabilizing. Additionally, Acharya et al. performed single amino-acid substitutions in the basic leucine zipper protein VBP and found the same trend previously observed by the group of Hodges.^15,16^ Substitution of asparagine in the hydrophobic core has also been investigated, as it is a common component of natural coiled-coil peptides and is useful for controlling peptide oligomerization, although at the cost of the overall stability of the coiled coil.^17,18^

The development of added functionality has mostly focused on responses of core modification to an external trigger. For example, redox switching of methionine residues in the hydrophobic core was found to result in disruption of the coiled coil.^19^ In addition, substitution of hydrophobic amino acids for histidine residues leads to self-assembly, which is dependent on pH or metal ion coordination.^20−22^ These studies show the ability of the hydrophobic core to adapt to different amino acids with different sizes and polarities. Therefore, the incorporation of photoswitchable azobenzene-based amino acids, which change structure and polarity through cis/trans isomerization,^23^ should allow for photocontrol over coiled-coil assembly. Azobenzene motifs are well known for acting as biomolecular switches, and their utility has been extensively reviewed.^24−26^

**Figure 1:**
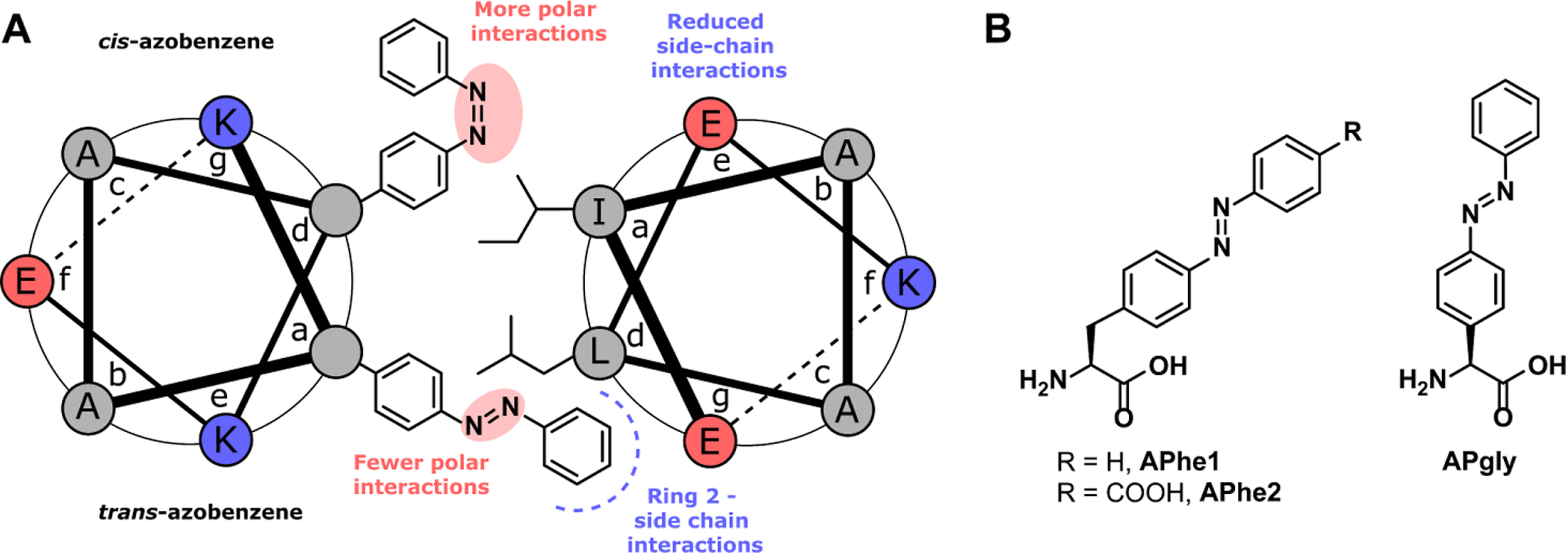
(A) Hypothesized interactions of azobenzene in the coiled-coil hydrophobic core. (B) The three azobenzene-based amino acids investigated for control over coiled-coil self-assembly.

Phenylazo-phenylalanine (**APhe1**, Figure 1) was one of the first published amino acids containing an azobenzene moiety^27^ and has been incorporated into synthetic peptides and into protein structures via genetic code expansion.^28^**APhe1** and structural derivatives have been used in a variety of applications, for example, to control enzyme dimerization necessary for catalytic activity^29,30^ and to alter the DNA binding strength of the CAP transcription factor by destabilization of its cAMP binding site,^28^ by incorporation into superfolder green fluorescent protein,^31^ and allosterically reducing the protein–ligand binding strength in chemiluminescent luciferase or subunit interactions in imidazole glycerol phosphate synthase.^32,33^ Derivatives of **APhe1**-containing reactive sites for the generation of intramolecularly cross-linked proteins have also been studied^34,35^ and allow for control over helical folding in a manner similar to the cross-linking strategy discussed previously. Since straightforward methodologies have been developed for the incorporation of **Aphe1** into peptides, its ability to control coiled-coil assembly seems like a logical next step toward controllable photoswitching of coiled-coil systems already under development for biomedical applications.^10,11^

To test whether azobenzene-based amino acids can indeed be used to control coiled-coil folding, peptides incorporating three non-natural amino acids were prepared by solid-phase peptide synthesis (SPPS). Two of the amino acids (**Aphe1** and **Aphe2**; Figure 1B) are derived from l-phenylalanine, whereas the third (**APgly**) is based on phenylglycine. The difference between **APhe1** and **APhe2** exists in the presence of a carboxylic acid group in **APhe2** located on the para-position of the terminal phenyl ring. The envisioned function of this carboxylic acid group is to introduce electrostatic repulsion between itself and the glutamic acid residues in the opposing peptide. This is expected to destabilize coiled-coil assembly upon switching of the azobenzene to the trans conformation. This concept of switching electrostatic interactions has previously been investigated via enzymatic serine phosphorylation^36^ and allowed both stronger or weaker interactions after phosphorylation, depending on the design.^3^ The l-phenylglycine derivative, **APgly**, lacks a methylene group in comparison to **APhe1,** which reduces the size of the amino acid. The smaller size of this amino acid is expected to be more suited for incorporation into the hydrophobic core of a coiled-coil motif, although its incorporation is not trivial as no synthetic strategy for its preparation is known.

The three azobenzene-based amino acids were incorporated into a dimeric coiled coil, which has been studied extensively as a SNARE protein mimic.^37^ This coiled-coil system consists of peptide “K_3_”, (KIAALKE)3, and peptide “E_3_”, (EIAALEK)3, which self-assemble into a dimeric parallel coiled coil with high binding affinity.^38^ Incorporation of **APgly** into peptide K_3_ showed the largest difference in coiled-coil binding upon trans-to-cis isomerization, and molecular dynamics (MD) simulations of **APhe1** and **APgly** incorporated into the coiled coil showed more rearrangement of the **APhe1** azobenzene after isomerization, therefore supporting **APgly** as the best molecular switch for coiled-coil photocontrol.

## Results and Discussion

### Peptides Containing Azobenzene Derivatives of Phenylalanine

**Scheme 1:**
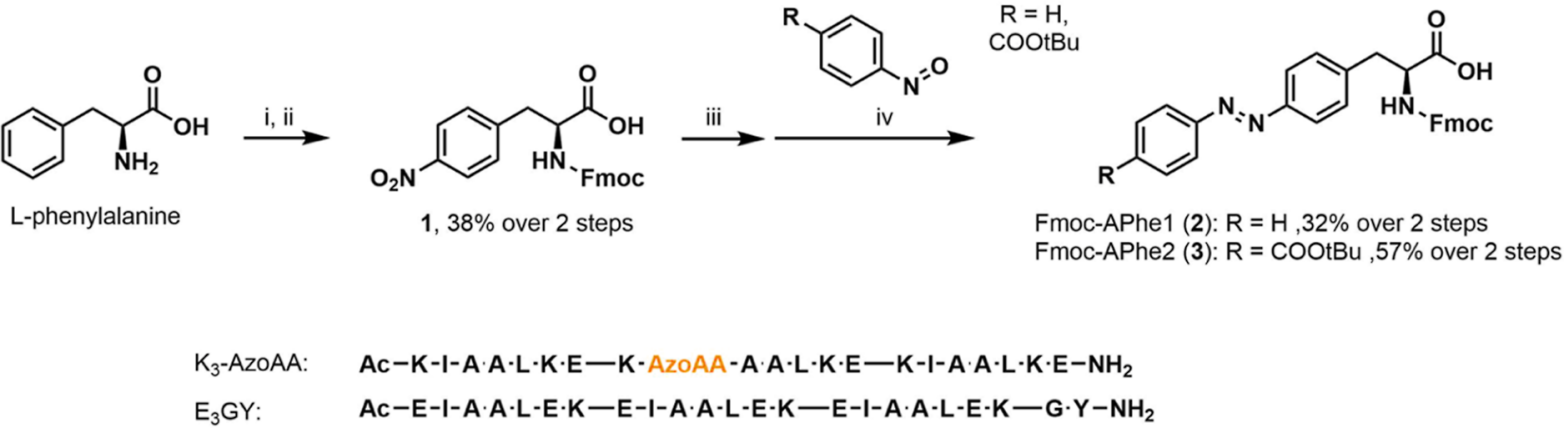
Reaction Scheme for the Preparation of Azobenzene-Containing Amino Acids Based on Phenylalanine (Top) and Amino Acid Sequences of Peptides Prepared in This Project (Bottom). Peptide E_3_ was Extended with Gly–Tyr at the C-Terminus to Facilitate Concentration Determination. AzoAA Refers to **APhe1**, **Aphe2,** or **APgly** Reagents and conditions: (i) HNO_3_, H_2_SO_4_, 0 °C to RT; (ii) Fmoc-chloride, NaHCO_3_, RT, 30% over 2 steps; (iii) Zn powder, NH_4_Cl, EtOH, reflux; (iv) AcOH, RT.

Photoswitchable amino acids based on phenylalanine were prepared following literature procedures (Scheme 1).^29^ In brief, Fmoc-4-nitro-l-phenylalanine, **1**, was prepared in 38% yield from l-phenylalanine via nitration and protection with a 9-fluorenylmethoxy-carbonyl (Fmoc) group or was purchased from a commercial source. Subsequently, the nitro group of **1** was reduced to aniline with Zn powder and ammonium chloride, followed by a Mills reaction with nitrosobenzene to yield N^**α**^-fmoc-4-(phenylazo)-l-phenylalanine **2** in 32% yield. The same procedure was followed for the preparation of Fmoc-protected **APhe2**, utilizing *tert*-butyl-4-nitrosobenzoate in the Mills reaction, resulting in product **3** in 57% yield. The *tert*-butyl ester is used to prevent side reactions during SPPS and is removed under acidic peptide cleavage conditions to yield carboxylic acid.

**Figure 2:**
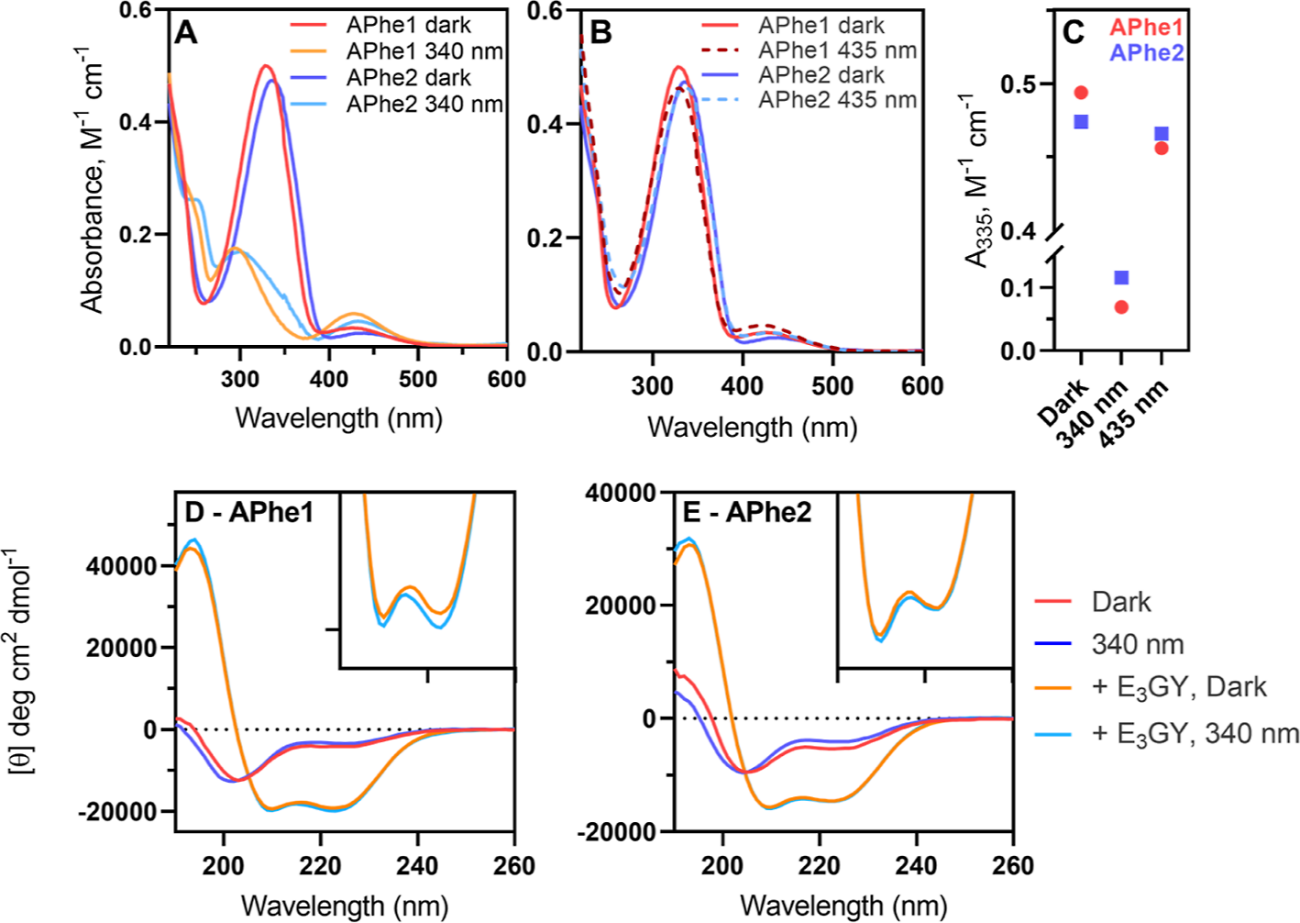
UV–vis (top) and CD (bottom) spectra of peptide K_3_ with position 9 substituted for **APhe1** or **APhe2**, showing the effect of illumination on azobenzene isomerization and peptide structures. (A) Absorbance spectra of peptides in the dark and after isomerization with 340 nm light to predominantly form the cis isomer. (B) Isomerization back from cis to trans using 435 nm light. (C) Overview of the absorbance at 335 nm for the dark-adapted, 340 nm PSS and 435 nm PSS for both peptides. (D) Effect of photoisomerization on the CD spectra of **K**_**3**_**-APhe1** by itself and as a coiled coil with E_3_GY. (E) Effect of photoisomerization on the CD spectra of **K**_**3**_**-APhe2** by itself and as coiled coil with E_3_GY. UV–vis samples were measured in PBS and CD samples in 10 mM phosphate buffer containing 2 mM NaCl to increase signals at lower wavelengths, with [peptide] = 50 μM.

Derivatives of peptide K_3_ (sequence shown in Scheme 1) were prepared via SPPS, with the isoleucine at position 9 replaced with **APhe1** or **APhe2.** The absorption spectra of the two peptides were very similar (Figure 2A), with a slight redshift for the absorption bands of **APhe2** (335 nm, ε = 9480 M^–1^ cm^–1^) compared to **APhe1** (327 nm, ε = 10,020 M^–1^ cm^–1^). Both amino acids can be isomerized from the trans to cis conformation with 340 nm light, as observed by the disappearance over time of the strong absorption bands at 335/327 nm respectively (Figure S1).

Cis to trans isomerization from the 340 nm photostationary state (PSS) could be achieved efficiently with 435 nm light (Figure 2B). The reduction in the observed absorption at 335 nm after isomerization with 340 nm light is larger for **APhe1**, indicating a larger amount of the cis isomer is present in the PSS for this amino acid (Figure 2C). Previously, a PSS of 79% (**APhe1**) or 81% (**APhe2**) cis isomer has been reported using a mercury lamp,^29^ or 89% *cis* for **APhe1** when using a LED light source.^33^ Cis to trans isomerization from the 340 nm PSS with 435 nm light is more efficient for **APhe2,** resulting in 98% of the absorption maximum observed in the dark state, compared to 92% for **APhe1**. The effect of phenylalanine-based photoswitches on peptide folding was subsequently studied by circular dichroism (CD) spectroscopy. Both **K**_**3**_**-APhe1** and **K**_**3**_**-APhe2** were mostly unstructured in solution and showed the formation of a helical structure when combined with E_3_GY (Figure 2D,E), with only marginal differences between the dark-adapted and 340 nm irradiated states.

As no obvious secondary structural differences were observed after irradiation, thermal denaturation experiments were performed to determine whether the binding affinity was dependent on sample irradiation. CD melting curves measured at different concentrations were fitted to a coiled-coil binding model (Figures S2 and S3).^39^ The best fit was achieved for a dimeric coiled-coil; thermodynamic parameters derived from this fitting are shown in Table 1. Photoisomerization of **K**_**3**_**-APhe1** using 340 nm light resulted in an increase in the unfolding constant *K*_u_ from 1.09 to 1.41 μM, showing reduced coiled-coil binding in the irradiated state. The opposite behavior was observed for **K**_**3**_**-APhe2**, where irradiation yielded a reduction in *K*_u_ from 4.58 to 3.07 μM, revealing that the 340 nm irradiated state contained the most stable coiled coil. The carboxylic acid group in **APhe2** was introduced to increase electrostatic repulsion with glutamic acid residues of E_3_ in the trans conformation, increasing the *K*_u_ of the dark state. Contrary to our expectations, however, the cis isomer of **K**_**3**_**-APhe1** was not the most stable state, demonstrating the bulk of the *trans* azobenzene moiety to be favored over the increased polarity of the diazene in the cis conformation.

**Table 1:** Fit Results of CD Thermal Unfolding Curves from **APhe1-** or **APhe2**-Containing K_3_ as a Coiled-Coil with E_3_GY^a^

	**K3-APhe1** dark	**K3-APhe1**340 nm	**K3-APhe2** dark	**K3-APhe2**340 nm
Δ*H*°^b^(kJ mol^–1^)	224.7	261.4	175.0	259.0
*T*°^b^(°C)	123	114.1	142.5	102.1
Δ*C*_p_^c^(kJ mol^–1^k^–1^)	1.69	2.46	1.07	2.65
*K*_f_(M^–1^)^d^	9.19 × 10^5^	7.09 × 10^5^	2.18 × 10^5^	3.26 × 10^5^
*K*_u_(μM)^d^	1.09	1.41	4.58	3.07
dark/340 nm PSS		1.30		0.67

a A complete list of fitting parameters and confidence intervals can be found in Table S1. The azobenzene amino acids are predominantly the trans isomer in the dark state and cis isomer in the 340 nm irradiated state.

b Δ*H*° and *T*° are the enthalpy and the temperature, where Δ*G* = 0 and *K*_u_ = *K*_f_ = 1.

c Δ*C*_P_ is the change in heat capacity upon unfolding.

d Binding model at 20 (°C).

In summary, incorporation of **APhe1** and **APhe2** in the coiled-coil hydrophobic core was successful, but differences in coiled-coil formation between the dark state and 340 nm PSS were found to be small. Given the small differences observed for the peptides modified with azo derivatives of phenylalanine, a different amino acid structure was designed. Removal of the methylene group from **APhe1** was theorized to position the diazene closer to the side chain of hydrophobic amino acids in the “a” and “d” positions due to the smaller size of the amino acid. This closer positioning is expected to increase the effect of photoisomerization on coiled-coil binding strength. The amino acid 4-(azophenyl)-phenylglycine (**APgly**, Figure 1) was therefore chosen to test this theory.

### Synthesis and Characterization of 4-(Azophenyl)phenylglycine-Containing Peptides

**Scheme 2:**
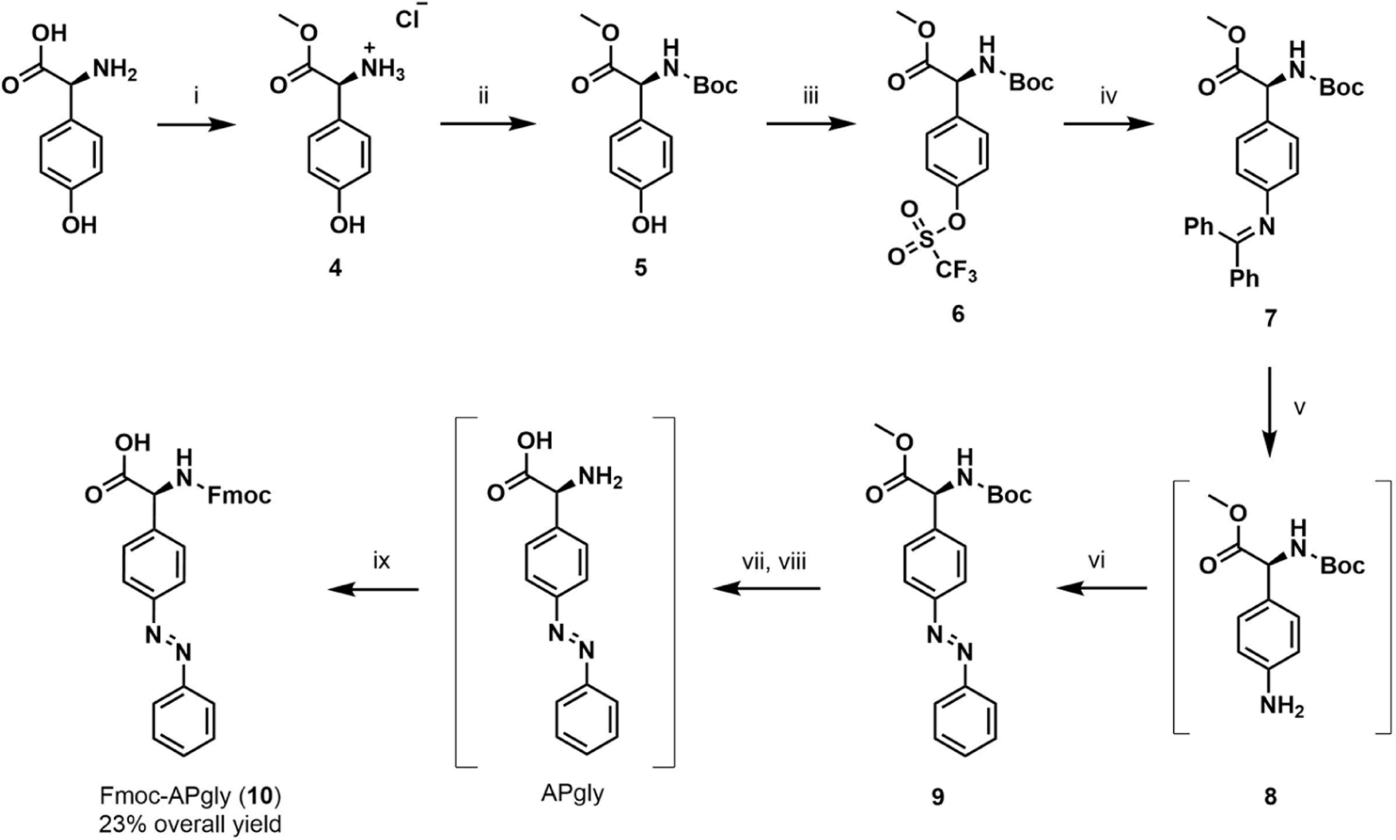
Synthetic Scheme for the Preparation of Fmoc-**APgly** from 4-Hydroxy-phenylglycine Reagents and conditions: (i) MeOH, SOCl_2_, 0 °C to RT, 95%; (ii) Boc_2_O, TEA, MeCN, RT, 93%; (iii) Tf_2_O, TEA, dichloromethane (DCM), −20 °C to RT, 98% (88% from 5); (iv) benzophenone imine, *rac*-BINAP, Pd(OAc)_2_, Cs_2_CO_3_, PhMe, 100 °C, 65%; (v) NH_4_HCO_2_, 10% Pd/C, MeOH, 60 °C; (iv) nitrosobenzene, AcOH, RT, 74% over 2 steps; (vii) LiOH, H_2_O/THF, RT; (viii) TFA, DCM, Rt; (ix) Fmoc-chloride, NaHCO_3_, H_2_O/THF, RT, 63% over 3 steps.

Synthesis of *N*-fmoc-4-(phenylazo)-l-phenylglycine (Fmoc-**APgly**, 10, Scheme 2) follows the same general route as the synthesis of azobenzene derivatives of phenylalanine; preparation of the para-substituted aniline, followed by a Mills reaction with nitrosobenzene to yield the azobenzene; preparation of the aniline required an alternative route, as nitration of phenylglycine does not yield the para-nitrated product; and preparation of the 4-nitro variant via a Strecker amino acid synthesis is not possible**.**^40^ Therefore, amination of the respective aryl triflate 6 was deemed the most effective route since the 4-hydroxy precursor is readily available. This route allowed access to **6** in sufficient quantities, from which **10** could be prepared following the procedure described below.

Methylation of 4-hydroxy-l-phenylglycine to form ester **4** was followed by amine protection with Boc anhydride to yield the double protected amino acid **5**, followed by installation of the aryl triflate to form intermediate **6** in high yields. Initially, the use of zinc trimethylsilylamide as an ammonia equivalent was attempted in the palladium-catalyzed amination of **6**,^41^ but the use of benzophenone imine proved much more effective, producing intermediate **7** in 65% yield.^42^ The imine was then reduced to yield aniline **8**, which was used without purification in a Mills reaction to form azobenzene **9**. Ester hydrolysis and Boc deprotection yielded **APgly**, which was Fmoc-protected to yield product **10**. The final three steps could be performed in good (63%) overall yield; however, attempts to simplify the procedure in the form of a one-pot procedure resulted in a sharp reduction in product yield. The overall yield of **10** from the starting material 4-hydroxy-l-phenylglycine is good (23% over 9 steps), although the atom efficiency could be improved given the large number of protecting group manipulations.

**Figure 3:**
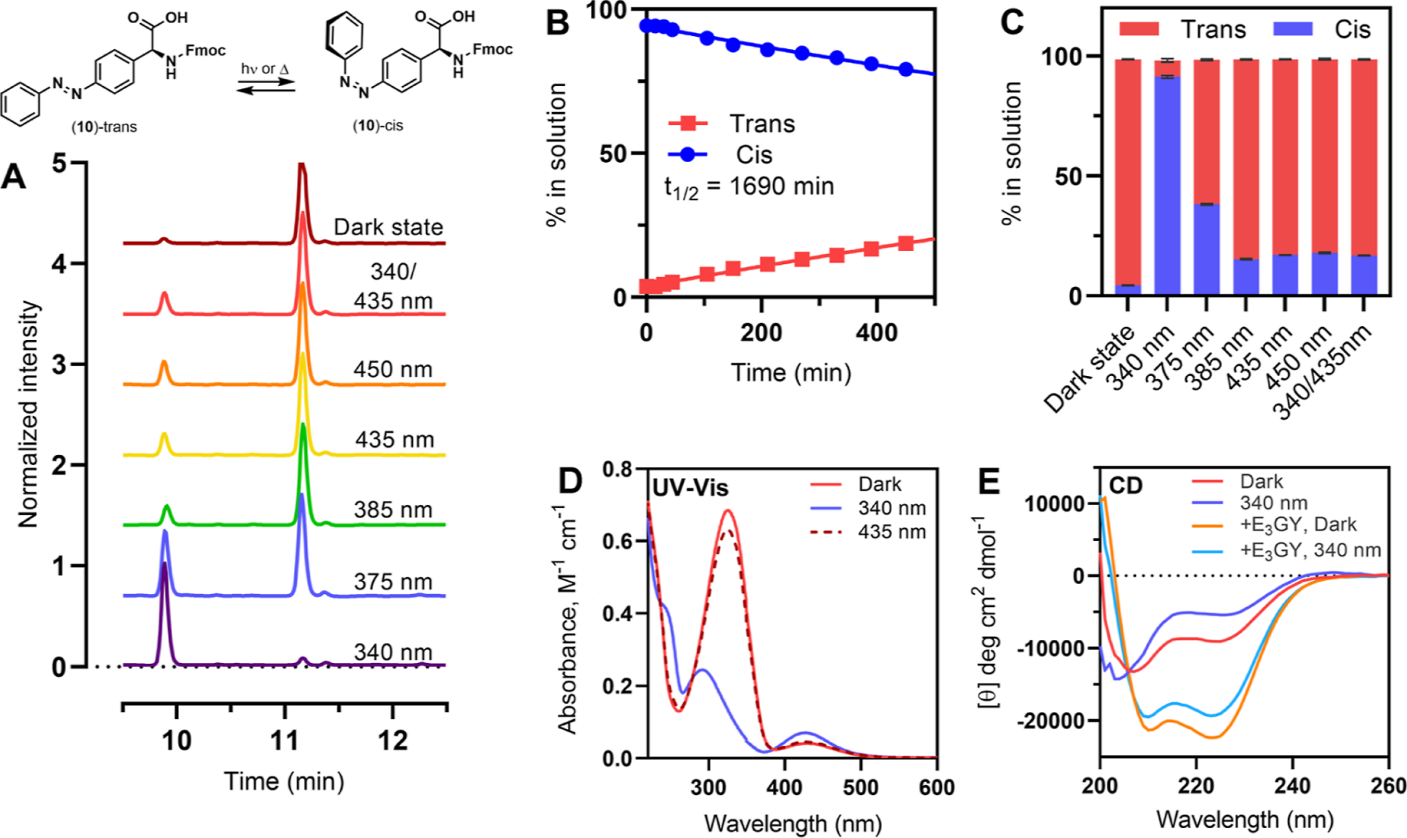
HPLC analysis of the photoswitching behavior of Fmoc-**APgly**, and the effect of azobenzene isomerization in peptide **K**_**3**_**-APgly**. (A) Chromatography traces of Fmoc-**APgly** in the dark state and after irradiation with different wavelengths of light, complete traces can be found in Figure S5. (B) Thermal relaxation of Fmoc-**APgly** from cis to trans as followed by HPLC. (C) Percentage of the trans and cis isomers in solution at the different PSSs; error bars indicate difference in deviation between repeat samples. (D) UV–vis spectra showing the spectra of **K**_**3**_**-APgly** in the dark and after irradiation with 340 or 435 nm light. (E) CD spectra showing the effect of photoisomerization on the structure of **K**_**3**_**-Apgly** by itself and as a coiled-coil with E3GY. Data labeled 340/435 nm are prepared by irradiating samples in the 340 nm PSS with 435 nm light. Illumination and relaxation studies of the amino acid were performed in MeCN at 20 °C. Peptide spectra were recorded at 20 °C in PBS with [peptide] = 50 μM.

As azobenzene photoswitches based on phenylglycine are not known in the literature, the photoswitching behavior of Fmoc-**APgly** (**10**) was analyzed first in order to assess its suitability as a photoswitch (Figure 3A–C). High-performance liquid chromatography (HPLC) separation of **10** dissolved in MeCN and kept under dark conditions showed predominantly the trans isomer (94.2 ± 0.2%), which switches to predominantly cis (91.3 ± 0.6%) when irradiated with 340 nm light (Figure 3C). The largest ratio of trans-to-cis isomers via photoisomerization was achieved through irradiation with 385 nm light (83.2 ± 0.2% trans), with higher wavelengths showing very similar distributions. No difference was observed in the proportions of isomers when **10** was directly illuminated with 435 nm light or first isomerized to be cis-dominant with 340 nm light, followed by illumination with 435 nm light, as is expected for the PSS.

Relaxation after irradiation with 340 nm light was tracked over time (Figure 3B) and showed a proportional decline of the cis isomer and increase of the trans isomer, with a half-life of 1690 min (≈28 h) comparable to reported half-lives for **APhe1** (1600 min) and **APhe2** (2100 min).^29^ Incorporation of **10** into K_3_ was achieved using normal Fmoc-SPPS methods. The trans isomer of **K**_**3**_**-APgly** showed a strong UV absorbance band at 325 nm (Figure 3D, ε = 13,700 M^–1^ cm^–1^) that disappeared upon irradiation with 340 nm light, accompanied by an increase in absorbance at 428 nm, demonstrating trans-to-cis isomerization. Irradiation of the sample with 435 nm light resulted in 92% of the dark absorbance at 325 nm, showing effective cis to trans isomerization. The observed absorbance values in UV–vis are in agreement with the isomeric ratios observed with HPLC demonstrating that **APgly** incorporation in peptides does not affect isomerization. CD spectra of **K**_**3**_**-APgly** showed a partially folded helix in the dark that became less structured when irradiated with 340 nm light (Figure 3E). The addition of the binding partner E_3_GY led to a well-folded coiled coil that also showed a reduction in signal upon isomerization. The difference in folding between dark-adapted and irradiated samples is much larger for peptide K_3_ containing **APgly** than it was for either of the phenylalanine derivatives, showing that the azobenzene is better positioned for coiled-coil photocontrol in the **APgly** derivative. The effect of isomerization on coiled-coil binding strength was again determined using thermal denaturation titration experiments (Figure S4, for fitting parameters see Tables 2 and S2). The coiled coil formed by **K**_**3**_**-APgly** and E_3_GY was determined to have a *K*_u_ of 0.72 μM in the dark, which increased to 3.37 μM after photoisomerization. This equates to a 4.65-fold reduction in binding affinity for **K**_**3**_**-APgly** by isomerization of the azobenzene to the cis-dominant state. The difference between the dark and 340 nm adapted state is larger for **K**_**3**_**-APgly** than was observed for either of the peptides that incorporated the phenylalanine derivatives, and the overall highest folding constant was also observed for this peptide. When comparing **APgly** isomerization to the amino acid substitution at position “a” studied by the group of Hodges,^13^ the change in binding energy is similar to substitution of alanine by a polar (Ser) or basic (Lys) side chain.

**Table 2:** Fit Results of CD Thermal Unfolding Curves for **K**_**3**_**-APgly** as a Coiled Coil with E3GY^a^

coiled-coil system	**K**_**3**_**-APgly** dark	**K**_**3**_**-APgly**340 nm
Δ*H*°^b^(kJ mol^–1^)	285.2	236
*T*°^c^(°C)	113.0	110.5
Δ*C*_p_(kJ mol^–1^k^–1^)	2.80	2.15
*K*_f_(M^–1^)^d^	1.38 × 10^6^	2.97 × 10^5^
*K*_u_(μM)^d^	0.72	3.37
dark/340 nm PSS		4.65

a A complete list of fitting parameters and confidence intervals can be found in Table S2. The azobenzene amino acids are predominantly the trans isomer in the dark state and the cis isomer in the 340 nm irradiated state.

b Δ*H*° and *T*° are the enthalpy and temperature, where Δ*G* = 0 and *K*_u_ = *K*_f_ = 1.

c Δ*C*_P_ is the change in heat capacity upon unfolding.

d Binding model at 20 °C.

**Figure 4:**
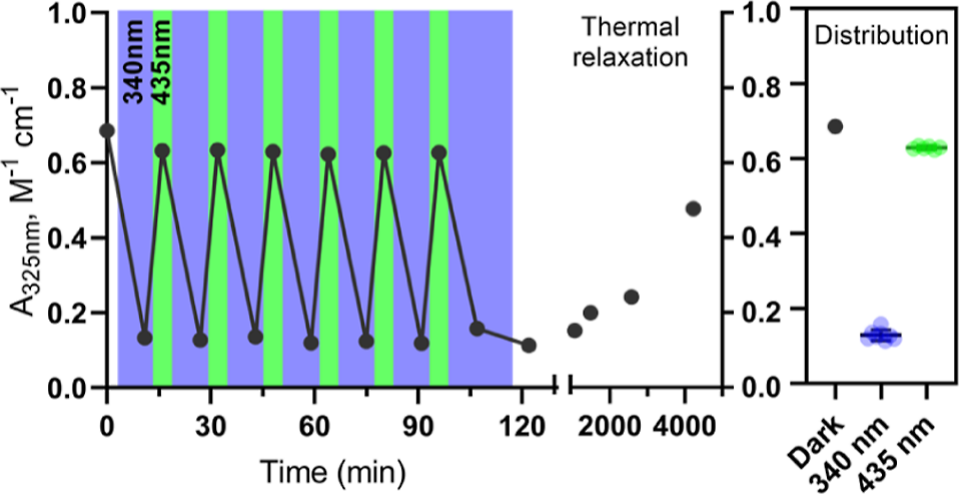
Photocycling of **K**_**3**_**-APgly** between the dark-adapted, 340 and 435 nm PSS (left), thermal relaxation of the peptide after thermal cycling (middle) and distribution of the absorbance in the different states during cycling (right). Absorbance at 325 nm is plotted (full spectra are displayed in Figure S6), and measurements were performed at 20 °C in PBS with [peptide] = 50 μM.

For the potential application of **K**_**3**_**-APgly** in an active system, for example, for membrane fusion, the stability of the photoswitch to repeated light exposures (photocycling) is required. To test the photostability of **K**_**3**_**-APgly**, solutions of this peptide were repeatedly illuminated with 340 and 435 nm light until the PSS was achieved, and the photoswitching was monitored via the UV absorbance band at 325 nm (Figures 4 and S6). The azobenzene consistently yielded the same absorption maxima over six cycles, demonstrating the cycling of PSSs with minimal deviation. Finally, the peptide was irradiated with the 340 nm PSS and allowed to thermally relax, showing 65% recovery in 69 h. This thermal relaxation is 1.6 times slower (*t*_1/2_ = 46 h) than that observed for **10** in MeCN, which was attributed to the larger dielectric constant of the solvent.^43^

### Molecular Simulations Illustrate the Difference between APhe and APgly

We have demonstrated that the K_3_/E_3_ coiled coil containing a single azobenzene-based amino acid can be switched between two different states most effectively when the photoswitch is derived from phenylglycine. To understand how this difference of one methylene group affects azobenzene interactions, molecular models of **K**_**3**_**-APgly** and **K**_**3**_**-APhe1** were prepared. These models were based on the NMR structure of the K_3_/E_3_ coiled-coil,^44^ with the Ile residue at position 9 replaced by a geometry optimized model of the synthetic azobenzene-based amino acids in the cis or trans conformations. 500 ns MD simulations of the K3/E3 coiled-coils with **APgly** and **APhe1** modifications show reasonably stable helical coiled-coil structures (Figures S7 and S8), although some unfolding at the termini was observed.

**Figure 5:**
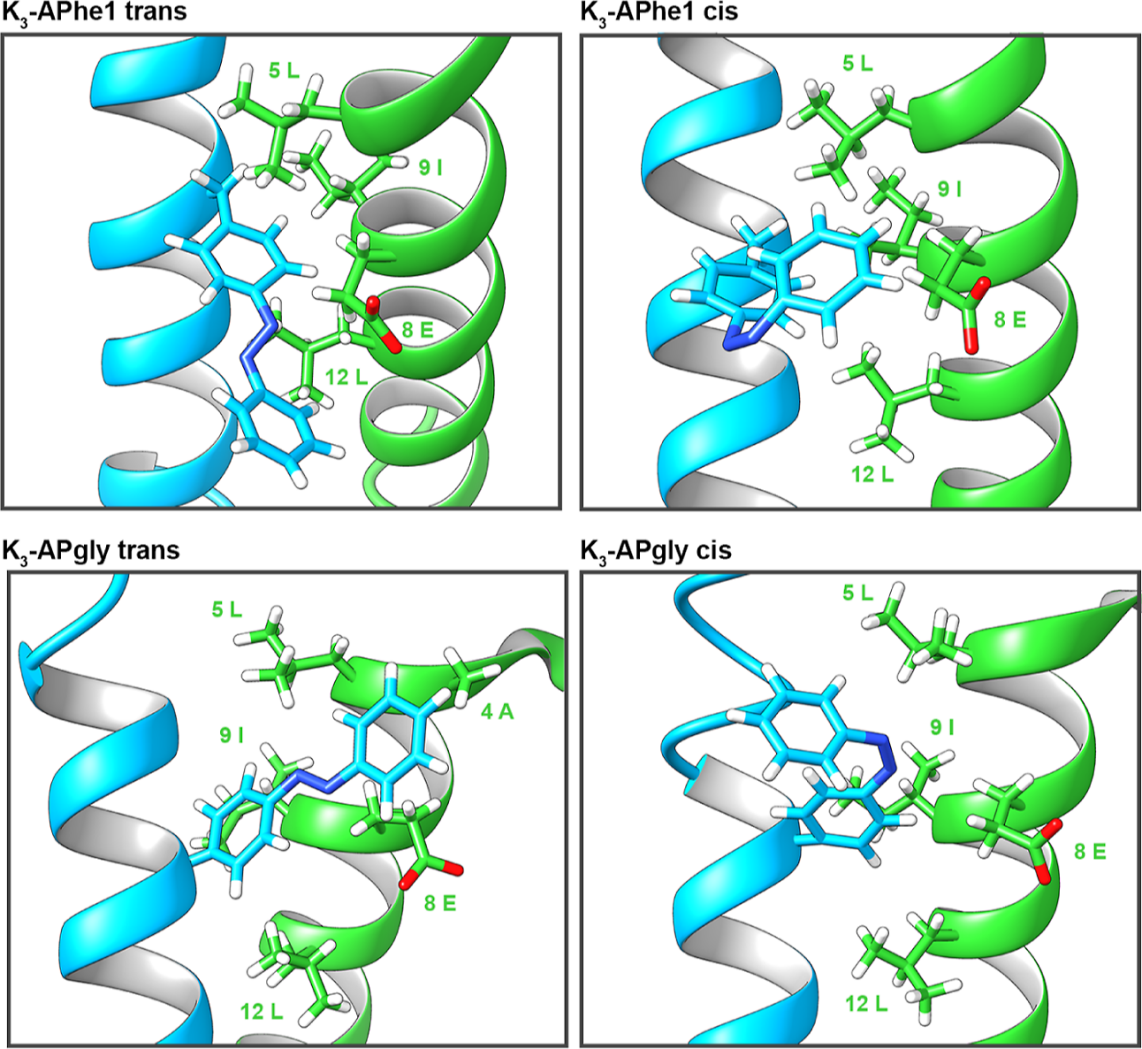
Snapshots from MD simulations of the coiled coil formed between peptide K_3_ (blue) and E_3_ (green), with peptide K3 containing the photoswitchable amino acid **APhe1** or **APgly** in the trans or cis conformation. Peptide backbones are shown as a cartoon, with the photoswitch and all amino acid side chains of peptide E3 within 5 Å displayed as sticks. Representative snapshots were chosen based on a similar rmsd from an ideal helix. Complete images are shown in Figure S9.

Snapshots from these MD simulations, displaying the azobenzene-amino acid and all side chains of peptide E_3_ within 5 Å of that residue, are shown in Figure 5. For both peptides, azobenzene is shown to be close to the hydrophobic amino acids (Leu5, Ile9, and Leu12) in the “a” and “d” positions, as well as adjacent amino acids (Ala4 and Glu8) in “g” and “c” positions. The outer phenyl groups of both azobenzene amino acids extend outside of the hydrophobic core (as viewed from the backbone and referred to as “Ring 2”). Therefore, the shielding of the azobenzene ring 2 from the surrounding water by amino-acid side chains will have an impact on coiled-coil stability. The snapshot displays the diazo group of *cis***K**_**3**_**-APgly** in the same position as in the *trans* conformation, with ring 2 rotated toward peptide K. The opposite is observed in the snapshots of **K**_**3**_**-APhe1**, where the diazene group is in a different position relative to K_3_, and ring 2 is still able to interact with amino acids in peptide E_3_.

**Figure 6:**
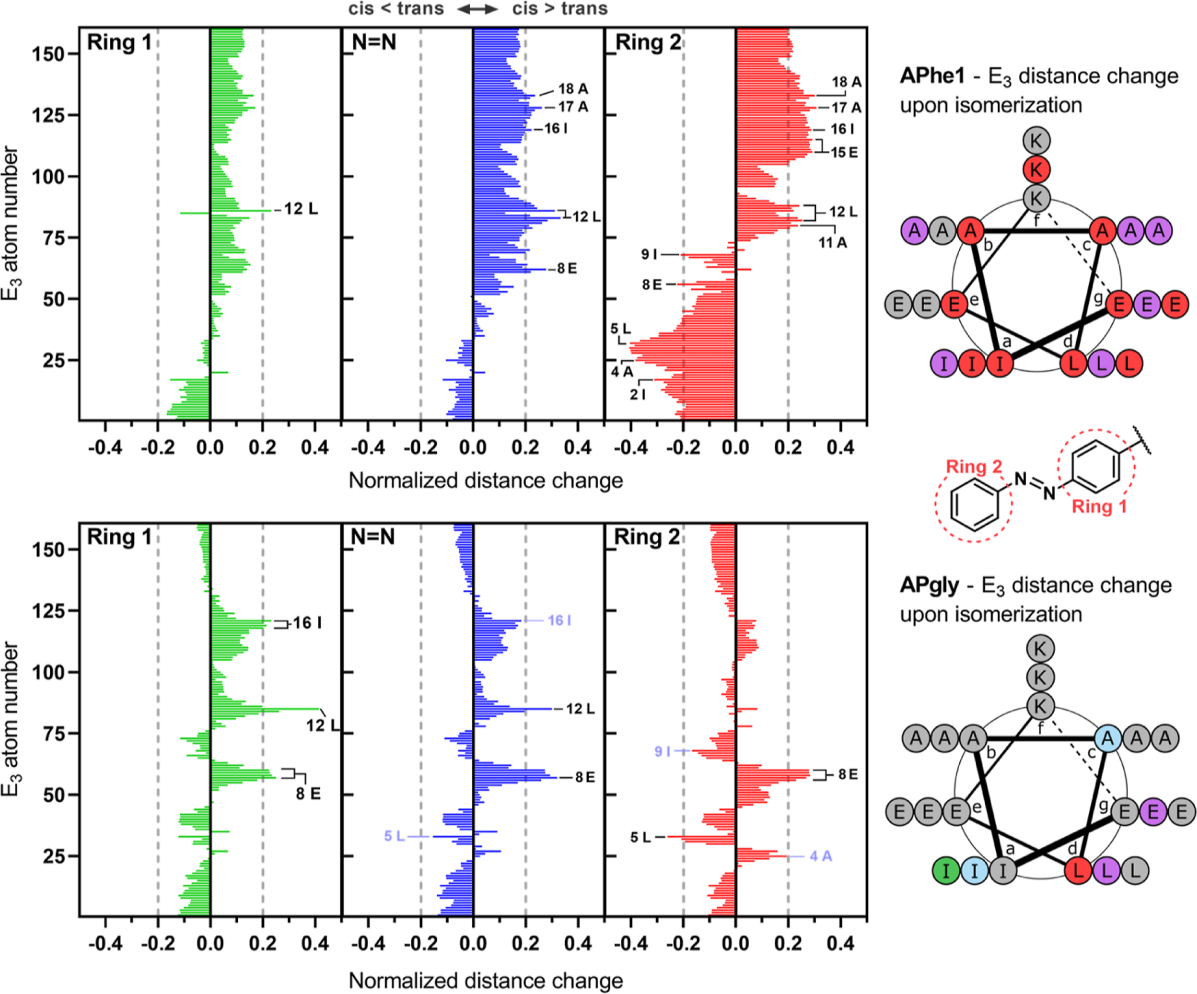
Normalized changes in distance upon photoisomerization between the non-hydrogen atoms in peptide E_3_ and the azobenzene moieties during MD simulations (left), and helical wheel diagrams of peptide E_3_ indicating the positions of the amino acids with significant changes in distance (right). Change in distance upon trans-to-cis isomerization of the azobenzene is normalized to trans simulations, with positive values indicating more distance in the cis simulations. Distances are averaged over three simulations of 500 ns. Dotted lines indicate an arbitrary cut-off for significance of 0.2, with amino acid labels of interest marked light blue if their value falls below this line. Amino acids in the helical wheel diagram are colored to indicate significant differences to the diazo group (blue), ring 1 (green), ring 2 (red), or multiple (purple), with amino acids of interest, which fall below the cut-off and are also colored light blue.

In order to study the dynamics of the system, the average change in distance upon photoisomerization between the azobenzene and the amino acids of E_3_ was calculated over the full simulation trajectories (Figure 6). Distance changes to diazene, ring 1, and ring 2 were plotted separately to highlight the different effects of photoisomerization on these groups. Both the diazene and ring 2 of **APhe1** show a negative distance change for amino acids close to the C-terminus, indicating closer positioning to those groups in the cis conformation, which changes to positive values when moving through the sequence. A normalized change in distance of ≤−0.2 or ≥0.2 was deemed significant in order to compare different groups and photoswitches. The most significant distance changes were observed for ring 2 of **APhe1**, which is expected since it is further away from all points of rotation. All amino acids in the “a”, “c”, “d”, and “g” positions of E_3_ show significant changes in distance to **APhe1**, with the other three positions also showing some significant changes. The apparent cross-over point observed in the distance change graphs, combined with significant changes in distance upon **APhe1** isomerization for nearly all amino acids, suggests reorganization after isomerization, whereby the azobenzene can move to a different conformation to accommodate the cis isomer. This is supported by the distance change for ring 1, which shows the same general distribution of distance change throughout the sequence.

Peptide **K**_**3**_**-APgly** shows the largest change in distance for Leu side chains in the “d” positions of the heptad repeat sequence of E_3_ and to the Glu side chain at position 8. There are more distinct changes in distances to side chains in the “a” and “c” positions, but these fall outside of the significance threshold that was used for comparison and have been marked with a lighter color in Figure 6. Isomerization to the cis conformer is expected to result in repulsion of the diazene by the hydrophobic side chains. This is indeed observed in the form of an increased distance for Leu12 and Glu8, which is directly opposite to azobenzene in the coiled coil (see Figure 5). After isomerization, ring 2 of **APgly** shows positioning close to the amino acid side chains of K_3_ (Figure S10), which stabilizes the cis conformation. Overall, isomerization of **K**_**3**_**-APgly** results in distance changes, with the amino acids already positioned in close proximity in the trans conformation, indicating that the changes in distance can be attributed to the rotation around the diazene bond after isomerization. If we generalize the structures in the MD snapshots in Figure 5 with the changes in distance shown in Figure 6, the effect of isomerization on the coiled-coil folding constant observed in the previous section can be explained. Upon trans-to-cis isomerization, the diazene group becomes more polar, resulting in unfavorable interactions if it is positioned in the hydrophobic core. This is the same for both azobenzene amino acids, with a general increase in distance to diazene observed for the cis isomer. **APhe1** has two bonds between C_α_ and the azobenzene moiety, which allows for more degrees of freedom to reposition the azobenzene after isomerization. In comparison, **APgly** can only change the position via a single rotation and through deviation from the optimal bond angles. The extra rotational freedom in **APhe1** results in a decreased effect of the diazene polarity on coiled-coil stability through repositioning of the azobenzene. This repositioning also allows ring 2 to maintain favorable interactions with multiple hydrophobic side chains of peptide E_3_, which is not possible for **K**_**3**_**-APgly**. Both of these factors decrease the differences in binding strength between the two isomeric states of **APhe1** and explain why **APgly** shows the largest difference in coiled-coil binding upon isomerization.

## Conclusions

Two azobenzene derivatives of phenylalanine, **APhe1** and **APhe2**, were prepared and incorporated into the hydrophobic core of peptide K_3_. Both azobenzene-containing peptides showed good photoswitching behavior, although azobenzene isomerization had only a moderate effect on coiled-coil interactions with the binding partner E_3_GY. A novel azobenzene amino acid, **APgly**, was subsequently prepared based on l-phenylglycine and showed comparable photoswitching characteristics to the phenylalanine-based azobenzene switches. When incorporated into peptide K_3_, **APgly** showed good stability to photocycling under aqueous conditions and exhibited a 1.6-fold slower relaxation from the 340 nm PSS compared to the protected amino acid. CD experiments demonstrated a difference in folding between the dark and irradiated states, and thermal melting experiments revealed a 4.65-fold reduction in the coiled-coil folding constant after isomerization with 340 nm light, combined with the highest overall folding in the dark state.

To better understand the interactions of the azobenzene amino acid in the coiled coil, MD simulations of **K**_**3**_**-APhe1** and **K**_**3**_**-APgly** with the binding partner E_3_ were performed. These simulations showed more distance changes between the cis and trans isomers of **APhe1** than were observed for **APgly**, indicative of a rearrangement of the amino acid after isomerization. Positioning of the **APgly** diazene group close to the center of the hydrophobic coiled-coil interface, combined with less rearrangement of the azobenzene after isomerization, makes **APgly** the more effective photoswitch for functional coiled-coil motifs and assemblies.

This work establishes **APgly** as a novel photoswitchable amino acid that can be used to control the activity of coiled-coil peptides through disruption of the hydrophobic core. Because azobenzene isomerization directly affects the binding interface, this method of photocontrol should extend to other coiled coils. Additionally, control over peptide self-assembly through photoswitchable amino acids is not limited to coiled coils but can be extended to other peptide structures with large hydrophobic domains, meaning such an approach has potentially wide-reaching applications in, for example, modulation of protein–protein interactions and the preparation of responsive biomaterials.

## Experimental Section

### General

Fmoc-protected amino acids and Fmoc chloride were purchased from Novabiochem (Amsterdam, The Netherlands). Acetic anhydride (Ac_2_O), acetonitrile (MeCN), dimethylformamide (DMF), piperidine, pyridine, NaHCO_3_, trifluoroacetic acid (TFA), and tetrahydrofuran (THF) were purchased from Biosolve (Valkenswaard, The Netherlands). Oxyma pure was purchased from Carl Roth (Karlsruhe, Germany). Acetic acid (AcOH), ammonia, ammonium chloride, ammonium formate, benzophenone imine, 2,2′-bis(diphenylphosphino)-1,1′-binaphthalene (*rac*-BINAP), 1,2-bis(2-mercapto-ethoxy)-ethane, cesium carbonate, di-*tert-*butyl decarbonate (Boc_2_O), Fmoc-4-nitro-l-phenylalanine, 4-hydroxy-l-phenylglycine, lithium hydroxide, nitrosobenzene, *N*–*N*′-diisopropylcarbodiimide (DIC), oxone, palladium acetate, palladium on carbon (Pd/C, 10%), l-phenylalanine, *tert*-butyl 4-aminobenzoate, thionyl chloride, triethylamine (TEA), trifluoromethanesulfonic anhydride (Tf_2_O), and zinc powder were purchased from Sigma-Aldrich (Zwijndrecht, The Netherlands). Chloroform, DCM, diethyl ether (Et_2_O), ethyl acetate (EtOAc), ethanol (EtOH), methanol (MeOH), pentane, petroleum ether (PE), sodium sulfate, and toluene were supplied by Honeywell (Meppel, The Netherlands). Tentagel S RAM resin was purchased from Rapp Polymere (Tübingen, Germany). All reagents were used as purchased. Ultrapure water was purified using a Milli-Q purification system from Millipore (Amsterdam, The Netherlands).

Peptide synthesis was performed via Fmoc-based SPPS on a CEM Liberty Blue microwave-accelerated peptide synthesizer. Peptides were prepared on a 0.1 mmol scale using Tentagel S RAM resin (0.22 mmol/g). 5 equiv each of amino acid, oxyma pure, and DIC were heated at 90 °C for 4 min to facilitate coupling. Deprotection was achieved with 20% piperidine in DMF heated to 90 °C for 1 min. Between deprotection and peptide coupling, three DMF washes were performed, with a single washing step between the coupling and deprotection steps. Azobenzene aminoacids **2**, **3,** and **10** were coupled manually using 2.5 equiv of amino acid, 2.5 equiv of HATU, and 5 equiv DIPEA in DMF for 2–3 h. After the last Fmoc deprotection, the N-terminus was acylated using 5 mL/mmol each of Ac_2_O and pyridine in DMF for 5 min. The resin was washed three times with DMF, MeOH, and DCM, followed by air-drying. Cleavage of peptide was achieved performed with TFA (8 mL) containing 2.5% water and 2.5% TIS for 1 h, followed by precipitation of the product in Et_2_O. The product was collected via centrifugation (4000 rpm, 10 min), the organic layer removed, and the product resuspended in water for direct purification or lyophilization.

Peptides were purified using reversed-phase HPLC on a Shimadzu system consisting of two KC-20AR pumps and an SPD-20A or SPD-M20A detector fitted with a 21.2 × 150 mm Phenomenex Kinetex Evo C18 column. A linear gradient from 10–90% MeCN in water was used, with 0.1% TFA as the ion-pair reagent at a flow rate of 12 mL/min. Collected fractions were checked via analytical HPLC, pooled, and lyophilized twice to yield the dry products. MS characterization of purified peptides can be found in Table S4.

### Analytical Methods

LC–MS analysis was performed on a Thermo Scientific TSQ quantum access MAX mass detector connected to an Ultimate 3000 liquid chromatography system fitted with a 50 × 4.6 mm Phenomenex Gemini 3 μm C18 column. LC–MS spectra were recorded using a linear gradient of 10–90% MeCN in H_2_O + 0.1% TFA.

Analytical HPLC was performed using a Shimadzu Prominence-*i* LC-2030C 3D system fitted with a 4.6 × 50 mm Phenomenex Kinetex Evo C18 column. The isomeric ratio of **10** was quantified using a linear gradient of 10–90% MeCN in water containing 0.1% TFA as buffer. For each measurement, 2 μL of a 200 mM solution of **10** was injected, and measurements were repeated three times for accuracy.

UV–vis spectra were measured on an Agilent Cary-300 spectrophotometer fitted with an Agilent temperature controller. Spectra were measured at 20 °C in a 1 cm quartz low-volume cuvette, using a scanning speed of 200 nm/min. Spectra were baseline corrected using a blank measurement with the same solvent used for sample preparation.

CD spectra were recorded on a Jasco J-815 CD spectrometer fitted with a Peltier temperature controller. Spectra were recorded in a 2 mm quartz cuvette at 20 °C using either PBS or low-salt buffer (2 mM NaCl, 10 mM phosphate) at pH 7.4. Spectra were recorded between 190 and 280 nm with 1 nm intervals at a scan rate of 100 nm/min with five subsequent spectra averaged to minimize noise. The mean residue molar ellipticity (θ, deg cm^2^ dmol res^–1^) was calculated using eq 1
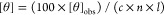
1where [θ]_obs_ represents the observed ellipticity in mdeg, *c* represents the peptide concentration in mM, *n* is the number of peptide bonds, and *l* is the path length of the cuvette in cm. Thermal melting curves were generated by recording θ_222nm_ between 5 and 90 °C at a speed of 1 °C/min. If irradiated samples were used, samples were reilluminated every 30 min. Melting curves were measured at four different concentrations and fitted using the Fitdis software package.^39^

Sample illumination at 340 nm was achieved using a Thorlabs M340F3 Fiber-Coupled LED powered by a T-Cube driver at 1000 mA. For all other wavelengths, high-power single-chip LEDs were purchased from Roithner Laser (Vienna, Austria) from the H2A1 series. LEDs from Roithner Laser were mounted on an aluminum back plate for heat dissipation and powered at 350 mA current using a driver built in-house. For illumination, the LED was placed parallel to the side of the cuvette at a distance of 5 mm and centered to the width and height of the sample.

### Molecular Simulations

#### Geometry Optimization

Input geometries of the amino acids for MD simulations were obtained by geometry optimization performed using the Amsterdam density functional (ADF2019.302)^45−47^ software package, using the BLYP functional with the D3(BJ) dispersion correction^48,49^ and a TZ2P basis set, which is of triple-ζ quality for all atoms and has been improved by two sets of polarization functions. The accuracies of the fit scheme (Zlm fit) and the integration grid (Becke grid) were set to VERYGOOD. All structures were verified to be at the stationary point by the absence of negative frequencies.

#### Force Field Parametrization

A new atom-type NX was introduced to the AMBER-96 force field^50^ for the diazo nitrogen atoms in **APgly** and **APhe1**. Bond lengths, angles, and dihedral angles for this new atom type were derived from DFT-calculated optimized geometries and are displayed in Table S3. Force constants from similar existing atom-type combinations were adopted. For the production runs, a high force constant (1000 kJ mol^–1^ rad^–2^) was used to restrain the cis/trans dihedral angle.

#### System Setup

The NMR structure of the E_3_/K_3_ coiled-coil heterodimer was retrieved from the protein data bank (PDB, ID: 1U0I).^44^ Ile9 of peptide K was mutated manually to **APgly** or **APhe1** and solvated with TIP3P water^51^ in a 5 × 5 × 5 nm^3^ simulation box. After the steepest descent of energy minimization, the system was equilibrated for 500 ps with and, subsequently, without positional restraints on the protein atoms. For each system, three independent production runs of 500 ns were performed with pseudorandom initial velocities.

#### Simulation Details

All MD simulations were performed with GROMACS 2019.3.^52^ A 1 fs time step was used. Constant temperature (300 K, τ_T_ = 0.1 ps) and pressure (1 bar, τ_P_ = 2 ps) were maintained using the velocity rescaling thermostat^53^ and the Berendsen barostat,^54^ respectively. The system’s compressibility was set to 4.5 × 10^–5^ bar^–1^. Neighbor lists were recalculated every 100 steps with a cut-off of 1 nm using the Verlet cut-off scheme.^55^ Particle-mesh Ewald^56^ electrostatics (0.11 nm grid) and van der Waals interactions are shifted such that they switch off at the cut-off distance (1 nm).

### Organic Synthesis

#### Protocol A, General Method for the Reduction of Nitrophenylalanine

Fmoc-4-nitro-l-phenylalanine was dissolved in absolute EtOH (150 mL/g), combined with ammonium chloride (5 equiv) and Zn dust (4 equiv), and the reaction refluxed for 2 h. After the evaporation of the solvent, the resulting solids were combined with EtOAc and excess 1 M HCl, the phases were separated, and the aqueous layer was extracted twice with ethyl acetate. The two organic layers were combined, washed with deionized water, and dried with anhydrous Na_2_SO_4_. The solvent was evaporated under reduced pressure, yielding Fmoc-(4-amino)-l-phenylalanine was used without further purification.

#### 4-*tert*-Butyl-nitrosobenzoate

*tert*-Butyl 4-amino benzoate (765 mg, 3.96 mmol) was dissolved in DCM (10 mL) and combined with oxone (1.26 g, 8.29 mmol) in H_2_O (13 mL), and the mixture was stirred vigorously under reflux for 20 h. The layers were separated, and the aqueous layer was extracted with DCM (20 mL). The combined organic layers were washed sequentially with 1 M HCl (10 mL), half-saturated NaHCO_3_ (10 mL) and brine (10 mL), and dried over Na_2_SO_4_; the solvent was removed to yield the crude product. The product was purified via column chromatography (Et_2_O/C_5_H_12_) to yield 220 mg of the product as bright green needles (1.06 mmol, 27%), which was used directly in the Mills reaction. ^**1**^**H NMR** (500 MHz, CDCl_3_): δ 8.23 (d, *J* = 8.7 Hz, 2H), 7.91 (d, 2H), 1.62 (s, 9H). ^**13**^**C NMR** (126 MHz, CDCl_3_): δ 164.78, 164.47, 137.28, 130.94, 130.65, 123.51, 120.47, 82.53, 28.25.

#### *N*-Fmoc-4-nitro-l-phenylalanine (**1**)

To a solution of l-phenylalanine (4.96 g, 30 mmol) dissolved in H_2_SO_4_ (95%, 22.5 mL) and cooled over ice was added 4.2 mL of nitrating solution (prepared by mixing 2.8 mL of 60% HNO_3_ and 2.2 mL of 95% H_2_SO_4_ on ice) dropwise over 3.5 h. The solution was neutralized with ammonia solution (25%) added dropwise until pH 6 was achieved, and a precipitate was observed. The precipitate was collected by filtration and washed with water (5 × 30 mL). After drying, 3.5 g (55%) of 4-nitrophenylalanine was collected as an off-white solid. This intermediate (1.05 g, 5.00 mmol) was dissolved in a mixture of aqueous Na_2_CO_3_ (0.5 M, 20 mL), acetone (13 mL), and deionized water (13 mL), together with dodecyl sulfate (49.6 mg) and the mixture was cooled with an ice bath. A solution of Fmoc-chloride (1.32 g, 5.1 mmol) in acetone (10 mL) was added dropwise to the reaction mixture, and stirring was continued overnight at room temperature. The reaction was quenched by dilution into cold water (300 mL) and addition of 2 M HCl (5 mL). The resulting precipitate was filtered and redissolved in 0.5 M Na_2_CO_3_ (100 mL). The mixture was allowed to stir at 70 °C for 1 h, after which the white precipitate was filtered off, and the filtrate was collected and acidified to pH < 3 with 2 M HCl. The resulting precipitate was collected and dried to yield 1.5 g of a white solid (69, 38% over two steps). ^**1**^**H NMR** (400 MHz, DMSO-*d*_6_) 8.15 (d, 2H), 7.89 (d, *J* = 7.6 Hz, 2H), 7.80 (d, *J* = 8.7 Hz, 1H), 7.62 (dd, *J* = 7.5, 2.7 Hz, 2H), 7.56 (d, *J* = 8.5 Hz, 2H), 7.41 (t, *J* = 7.4 Hz, 2H), 7.30 (q, *J* = 7.4 Hz, 2H), 4.32–4.13 (m, 4H), 3.25 (dd, *J* = 13.8, 4.4 Hz, 1H), 3.02 (dd, *J* = 13.8, 10.8 Hz, 1H). **LC–MS** RT = 7.57 min, *m*/*z* = 178.47 (calcd Fm^+^ = 179.09), 454.61 (calcd [**1** + Na^+^]^+^ = 455.12), 470.00 (calcd [**1** + K^+^]^+^ = 471.10).

#### Preparation of *N*-Fmoc-(4-phenylazo)-l-phenylalanine, Fmoc-APhe1 (**2**)

Fmoc-4-nitrophenylalanine (1.34 g, 3.11 mmol) was reduced according to protocol A, resulting in 1.04 g of crude Fmoc-4-aminophenylalanine. The intermediate was dissolved in glacial AcOH (60 mL) before nitrosobenzene (350 mg, 3.27 mmol, 1.25 equiv) dissolved in the AcOH (5 mL) was added, and the solution left for 20 h. The product was precipitated by the addition of the reaction mixture to H_2_O (250 mL) and collected via filtration. The crude product was redissolved in EtOAc, dried with Na_2_SO_4_, and filtered, and the solvent was evaporated. The solids were purified via column chromatography (dry loading, DCM/MeOH + 0.5% AcOH), and the removal of solvents yielded 478 mg (0.97 mmol, 32% over 2 steps) of pure product as a brown powder. ^**1**^**H NMR** (500 MHz, DMSO-*d*_6_) 7.89–7.85 (m, 4H), 7.82 (d, *J* = 8.3 Hz, 3H), 7.66–7.54 (m, 5H), 7.50 (d, *J* = 8.4 Hz, 2H), 7.38 (q, *J* = 7.9 Hz, 2H), 7.28 (dt, *J* = 14.9, 7.4 Hz, 2H), 4.30–4.24 (m, 1H), 4.21 (d, *J* = 6.1 Hz, 2H), 4.19–4.13 (m, 1H), 3.21 (dd, *J* = 13.8, 4.4 Hz, 1H), 2.99 (dd, *J* = 13.8, 10.7 Hz, 1H). ^**13**^**C NMR** (126 MHz, DMSO) 173.14, 155.96, 151.99, 150.65, 143.77, 143.71, 142.06, 140.69, 140.68, 131.39, 130.24, 129.48, 127.61, 127.59, 127.05, 125.25, 125.19, 122.47, 120.10, 65.61, 55.22, 46.57, 36.32. **LC–MS** RT = 7.85 (cis), 8.90 (trans) min, *m*/*z* = 178.54 (calcd Fm^+^ = 179.09), 513.31 (calcd [**2** + Na^+^]^+^ = 514.17).

#### Preparation of *N*-Fmoc-l-(4-(4′-tert-butoxycarbonyl)-phenylazo)phenylalanine, Fmoc-APhe2 (**3**)

Fmoc-(4-nitro)-l-phenylalanine (874 mg, 2.02 mmol) was reduced according to general protocol A to yield 732 mg of Fmoc-(4-amino)-l-phenylalanine. The solids were dissolved with heating in glacial AcOH (70 mL), and after cooling to room temperature, *tert*-butyl 4-nitrosobenzoate (560 mg, 2.71 mmol) dissolved in AcOH (5 mL) was added, and the reaction was stirred for 20 h. Another portion of nitrosobenzene (220 mg, 1.06 mmol) was added, and the reaction was stirred for another 20 h. The product was precipitated in H_2_O (250 mL), collected via filtration, and redissolved in acetone; the solvent was removed via rotary evaporation. The crude product was purified via column chromatography (PE/Et_2_O + 1% AcOH), and the solvent was removed to yield 675 mg (1.14 mmol, 56% over two steps) of the pure product as a red semi-crystalline material. ^**1**^**H NMR** (400 MHz, CDCl_3_): δ 8.12 (d, *J* = 8.5 Hz, 2H), 7.89 (dd, *J* = 11.5, 8.3 Hz, 4H), 7.76 (d, *J* = 7.6 Hz, 2H), 7.55 (d, *J* = 7.2 Hz, 2H), 7.39 (t, *J* = 7.4 Hz, 2H), 7.33–7.28 (m, 4H), 5.30 (d, *J* = 8.1 Hz, 1H), 4.83–4.71 (m, 1H), 4.51–4.47 (m, 1H), 4.39 (dd, *J* = 10.7, 6.8 Hz, 1H), 4.21 (t, *J* = 6.9 Hz, 1H), 3.26 (ddd, *J* = 45.1, 13.9, 5.8 Hz, 2H), 1.63 (s, 9H). ^**13**^**C NMR** (101 MHz, CDCl_3_): δ 177.31, 165.40, 155.83, 154.93, 151.86, 143.84, 143.70, 141.47, 139.65, 133.84, 130.56, 130.38, 127.90, 127.23, 125.17, 125.11, 123.52, 122.63, 120.16, 81.71, 67.20, 54.58, 47.25, 37.82, 28.33. **LC–MS** RT = 8.79 (cis), 9.81 (trans) min, *m*/*z* = 178.47 (calcd Fm^+^ = 179.09), 591.25 (calcd [**3** + H^+^]^+^ = 592.24), 613.35 (calcd [**3** + Na^+^]^+^ = 614.23).

#### 4-Hydroxy-l-phenylglycine Methyl Ester (**4**)

Thionyl chloride (13.0 mL, 184 mmol, 9 equiv) was added dropwise to a suspension of 4-hydroxy-l-phenylglycine (3.39 g, 20.3 mmol) in dry MeOH (100 mL) over the course of 30 min, making sure to keep the temperature at 20 °C. The clear solution was stirred overnight, and all of the liquid was evaporated under reduced pressure. The resulting oil was mixed with Et_2_O (50 mL) to yield an off-white precipitate, which was filtered and washed with Et_2_O (2 × 50 mL). The residue was dried under vacuum to yield 4.33 g of 4-hydroxy-l-phenylglycine methyl ester hydrochloride (19.9 mmol, 98%). ^**1**^**H NMR** (400 MHz, DMSO-*d*_6_): δ 9.98 (s, 1H), 8.98 (s, 3H), 7.28 (d, *J* = 8.6 Hz, 2H), 6.84 (d, *J* = 8.6 Hz, 2H), 5.09 (s, 1H), 3.69 (s, 3H), 3.38 (s, 1H). ^**13**^**C NMR** (101 MHz, DMSO-*d*_6_): δ 169.26, 158.60, 129.64, 122.61, 115.70, 54.90, 53.02.

#### *N*-Boc-4-hydroxy-l-phenylglycine Methyl Ester (**5**)

A solution was prepared from 4-hydroxy-l-phenylglycine methyl ester (**4**, 1.11 g of the HCl salt, 5.11 mmol) in dry MeCN (70 mL) containing TEA (0.75 mL 5.39 mmol, 1.06 equiv). Boc_2_O (1.30 g, 5.96 mmol, 1.17 equiv) was dissolved in dry MeCN (20 mL) and added dropwise to the phenylglycine solution; the reaction was stirred at room temperature overnight. The solvent was evaporated, and the remaining solids redissolved in DCM (50 mL), which was subsequently washed with 1 M H_3_PO_4_ (25 mL), H_2_O (50 mL), and brine (25 mL). The organic layer was dried with Na_2_SO_4_, and the solvent was evaporated. The crude product was purified via filtration through a plug of silica (eluent: EtOAc), and the solvent was removed to yield 1.33 g (4.71 mmol, 93%) of product as a white solid. ^**1**^**H NMR** (400 MHz, DMSO-*d*_6_): δ 9.50 (s, 1H), 7.62 (d, *J* = 7.9 Hz, 1H), 7.20–7.13 (m, 2H), 6.75–6.65 (m, 2H), 5.05 (d, *J* = 7.9 Hz, 1H), 3.59 (s, 3H), 1.38 (s, 9H). ^**13**^**C NMR** (101 MHz, DMSO-*d*_6_): δ 171.91, 157.25, 155.20, 129.10, 126.64, 115.19, 78.42, 57.10, 52.00, 28.19.

#### *N*-Boc-4-(trifluoromethanesulfonate)-l-phenylglycine Methyl Ester (**6**)

*N*-Boc-4-hydroxy-l-phenylglycine methyl ester (1.41 g, 5.02 mmol) was dissolved in DCM (25 mL) and combined with TEA (1 mL, 7.5 mmol, 1.5 equiv). The reaction was cooled to −20 °C, and Tf_2_O (0.83 mL, 4.92 mmol, 0.98 equiv) was added in portions. After 30 min, the reaction was allowed to warm up to room temperature and left to stir for 4 h. The reaction was diluted with DCM (20 mL) and washed with 0.5 M HCl (20 mL), H_2_O (20 mL), and brine (20 mL). The organic layer was dried over Na_2_SO_4_, the solvent was removed, and the product was purified over a silica column (Et_2_O/C_5_H_12_) to yield 1.825 g of a clear oil (4.42 mmol, 98% yield relative to Tf_2_O, 88% relative to **5**). The oil could be turned into a solid via dissolution in pentane and removal of the solvent. ^**1**^**H NMR** (500 MHz, CDCl_3_): δ 7.47 (d, *J* = 8.7 Hz, 2H), 7.30–7.23 (m, 2H), 5.77–5.61 (m, 1H), 5.37 (d, *J* = 7.2 Hz, 1H), 3.75 (s, 3H), 1.43 (s, 8H). ^**13**^**C NMR** (126 MHz, CDCl_3_): δ 170.93, 154.76, 149.45, 137.93, 129.17, 121.92, 118.25(d, *J*_*CF*_ = 161 Hz), 80.72, 56.89, 53.21, 28.40, 27.80. ^**19**^**F NMR** (471 MHz, CDCl_3_): δ −72.86.

#### *N*-Boc-4-((diphenylmethylene)-amino)-l-phenylglycine Methyl Ester (7)

An oven-dried Schlenk reaction vessel was charged with Cs_2_CO_3_ (460 mg, 1.4 mmol, 1.4 equiv), benzophenone imine (210 μL, 1.25 mmol, 1.21 equiv), and *N*-Boc-(4-trifluoromethanesulfonate)-l-phenylglycine methyl ester (**6**, 413 mg, 1.03 mmol), and the flask was placed under a nitrogen atmosphere. Pd(OAc)_2_ (15.5 mg, 0.07 mmol, 7%) and *rac-*BINAP (95 mg, 0.15 mmol, 15%) were combined in a separate vial, placed under nitrogen, combined with toluene (4 mL) and H_2_O (2.5 μL, 0.14 mmol, 14%), and heated to 85 °C. After 2 min, the color of the catalyst solution changed to bright red, and the solution was transferred to the reaction flask and the reaction was stirred at 100 °C for 20 h. After cooling to RT, the liquid phase of the reaction was transferred to a separatory funnel, the solids were washed with Et_2_O (3 × 10 mL), and this liquid was also combined in the funnel. The organic layers were extracted with H_2_O (2 × 20 mL) and dried with Na_2_SO_4_, and the solvent was removed. The product was purified over the silica column (Et_2_O/C_5_H_12_) and dried under high vacuum to yield 296 mg of a light-yellow solid (0.66 mmol, 65% yield). ^**1**^**H NMR** (500 MHz, CDCl_3_): δ 7.72 (d, *J* = 7.0 Hz, 2H), 7.46 (t, *J* = 7.3 Hz, 1H), 7.39 (dd, *J* = 8.3, 6.8 Hz, 2H), 7.25 (d, *J* = 8.1 Hz, 4H), 7.14–7.07 (m, 4H), 6.69 (d, *J* = 8.4 Hz, 2H), 5.39 (d, *J* = 7.5 Hz, 1H), 5.19 (d, *J* = 7.5 Hz, 1H), 3.68 (s, 3H), 1.43 (s, 8H). ^**13**^**C NMR** (126 MHz, CDCl_3_): δ 171.96, 168.66, 154.95, 151.54, 139.62, 136.07, 131.34, 130.97, 129.61, 129.47, 128.83, 128.34, 128.09, 127.56, 121.53, 80.21, 57.33, 52.65, 28.42.

#### *N*-Boc-(4-phenylazo)-l-phenylglycine Methyl Ester (**9**)

Ammonium formate (1.1 g, 17.5 mmol, 15.8 equiv), 10% Pd on carbon (50% water by weight, 125 mg, 5.8%), and *N*-Boc-4-((diphenylmethylene)amino)-l-phenylglycine methyl ester (**7**, 496 mg, 1.11 mmol) were dissolved in MeOH (4 mL) under a nitrogen atmosphere, and the reaction was heated to 60 °C for 2 h. The reaction was diluted with DCM (10 mL), filtered over Celite, and again washed with DCM (20 mL). The organic layers were washed with H_2_O (2 × 30 mL) and brine (1 × 10 mL) and dried over Na_2_SO_4._ The solvent was removed to yield 400 mg of an off-white solid. This was redissolved in AcOH (5 mL), nitrosobenzene (167 mg, 1.56 mmol, 1.4 equiv) in AcOH (3 mL) was added, and the reaction was stirred for 6 days. DCM (50 mL) was added, and the organic layer was washed with H_2_O (50 mL), 3% NH_3_ (50 mL), and brine (50 mL). The organic layer was dried over Na_2_SO_4_, and the solvent was removed. The crude product was purified via column chromatography (Et_2_O/C_5_H_12_) to yield 303 mg of an orange solid (0.82 mmol, 74%). ^**1**^**H NMR** (400 MHz, CDCl_3_): δ 7.94–7.88 (m, 4H), 7.52 (m, 5H), 5.68 (d, *J* = 7.3 Hz, 1H), 5.41 (d, *J* = 7.3 Hz, 1H), 3.74 (s, 3H), 1.44 (s, 8H). ^**13**^**C NMR** (101 MHz, CDCl_3_): δ 171.29, 152.73, 152.66, 139.84, 131.33, 129.25, 128.05, 123.45, 123.05, 80.51, 57.48, 53.06, 28.45.

#### *N*-Fmoc-4-(phenylazo)-l-phenylglycine, Fmoc-APgly (**10**)

To *N*-Boc-4-(phenylazo)-l-phenylglycine methyl ester (**9**, 311 mg, 0.84 mmol) dissolved in THF (6 mL) was added 1 M LiOH (1.26 mL, 1.5 equiv), and the reaction was stirred for 90 min. After cooling, the reaction was combined with DCM (50 mL) and 0.5 M HCl (50 mL). The layers were separated, and the organic layer was washed with H_2_O (50 mL) and brine (50 mL) and dried over Na_2_SO_4_. The solvent was evaporated, the intermediate was redissolved in DCM (5 mL), and TFA (5 mL) was added. The reaction was stirred for 2 h, after which the intermediate was precipitated in cold Et_2_O/C_5_H_12_ (1:1; 100 mL) and collected via centrifugation at 4000 rpm for 10 min. The precipitate was dried under a stream of air for 5 min and then suspended in H_2_O/THF (1:3, 150 mL). NaHCO_3_ (1.5 g) was added to neutralize the solution, and Fmoc-chloride (258 mg, 1 mmol, 1.19 equiv) dissolved in THF (6 mL) was added to the reaction dropwise. The solution was stirred overnight, and the next day the THF evaporated. The reaction was combined with DCM (60 mL) and 1 M HCl (100 mL) and separated. The aqueous layer was extracted with DCM (40 mL), and the organic layers were combined and washed with H_2_O (100 mL) and brine (50 mL) and dried over Na_2_SO_4_. The solvent was removed, and the remaining solids were purified via column chromatography (DCM/MeOH with 0.5% AcOH) to yield 254 mg of product as a bright orange powder (0.3 mmol, 63% over 3 steps). ^**1**^**H NMR** (600 MHz, DMSO-*d*_6_): δ 13.11 (s, 1H), 8.44–8.31 (m, 1H), 7.93–7.87 (m, 6H), 7.77 (d, *J* = 7.5 Hz, 2H), 7.66 (d, *J* = 8.5 Hz, 2H), 7.64–7.55 (m, 3H), 7.44–7.39 (m, 2H), 7.32 (dt, *J* = 11.7, 7.5 Hz, 2H), 5.32 (d, *J* = 8.2 Hz, 1H), 4.34–4.22 (m, 3H). ^**13**^**C NMR** (151 MHz, DMSO-*d*_6_): δ 171.68, 155.91, 151.92, 151.50, 143.84, 143.77, 140.74, 140.66, 131.71, 129.55, 128.99, 127.70, 127.11, 125.42, 125.42, 122.64, 120.15, 66.01, 57.78, 46.62. **LC–MS** RT = 6.65 (cis), 8.80 (trans) min, *m*/*z* = 178.54 (calcd Fm^+^ = 179.09), 238.71 (calcd [**10** + 2H^+^]^2+^ = 239.59), 477.28 (calcd [**10** + H^+^]^+^ = 478.17), 499.46 (calcd [**10** + Na^+^]^+^ = 500.16).

## Supporting Information Available

Extra UV–vis, CD, and HPLC spectra as mentioned in the main text; detailed tables of fitting parameters used to calculate peptide binding; parameters used for MD simulations; different representations of coiled-coil structure during MD simulations; and full characterization of compounds prepared in this project (PDF)
