# Supplementary material for: Azobenzene-Based Amino Acids for the Photocontrol of Coiled-Coil Peptides
Source: Bioconjug Chem. 2023 Jan 27;34(2):345–57. doi: 10.1021/acs.bioconjchem.2c00534 (PMC9936496; doi:10.1021/acs.bioconjchem.2c00534)
Supplement: Supplementary file 1 — bc2c00534_si_001.pdf [file bc2c00534_si_001.pdf]

Supplementary information

Azobenzene-based amino acids for photocontrol of coiled coil peptides.

Niek S. A. Crone, Niek v. Hilten, Alex v. d. Ham, Herre Jelger Risselada, Alexander Kros* and Aimee L. Boyle*

Leiden Institute of Chemistry, Leiden University, Einsteinweg 55, 2333 CC, Leiden, The Netherlands.

*Corresponding authors. Alexander Kros: Leiden Institute of Chemistry, Leiden University, Einsteinweg 55, 2333 CC, Leiden, The Netherlands. Email: a.kros@chem.leidenuniv.nl

Aimee Boyle: Leiden Institute of Chemistry, Leiden University, Einsteinweg 55, 2333 CC, Leiden, The Netherlands. Email: a.l.boyle@chem.leidenuniv.nl

Table of contents

UV-VIS Spectra of K_3 Peptides with APhe1 or APhe2 2
Thermal unfolding curves of Photoswitchable coiled-coils 3
Chromatogrpahy traces of Fmoc-APgly 8
Photocycling of K_3-APgly 10
MD simulations extra figures 10
LC-MS Spectra of purified peptides 14
LC-MS Spectra of synthetic amino acids and building blocks 19
NMR Spectra of synthetic amino acids and building blocks. 23

UV-VIS spectra of K_3 peptides with APhe1 or APhe2

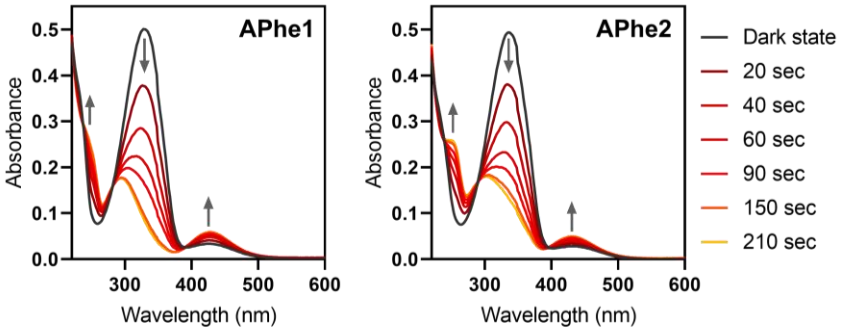

Figure S1: UV-Vis spectra of peptide K_3 containing either **APhe1** or **APhe2** at different time intervals during illumination with 340 nm light. Measurements were performed at 20 °C in PBS with [peptide] = 50 µM. Arrows indicate direction of change after illumination.

Thermal unfolding curves of photoswitchable coiled-coils

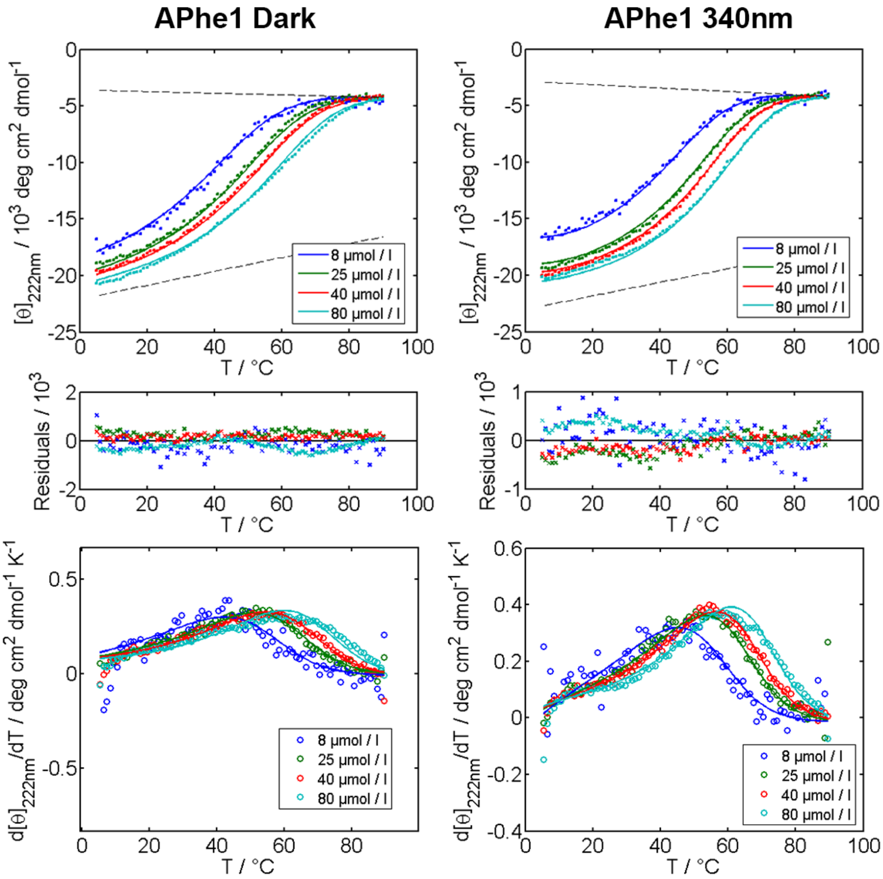

Figure S2: Fitting of CD melting curves (top) for peptide **K_3-APhe1** with E_3GY in the dark adapted (left) and 340 nm irradiated (right) states performed using the FitDis! software program. Residuals (middle) and derivatives (bottom) of the best fitting (dimeric) model are shown. Measurements were performed in PBS.

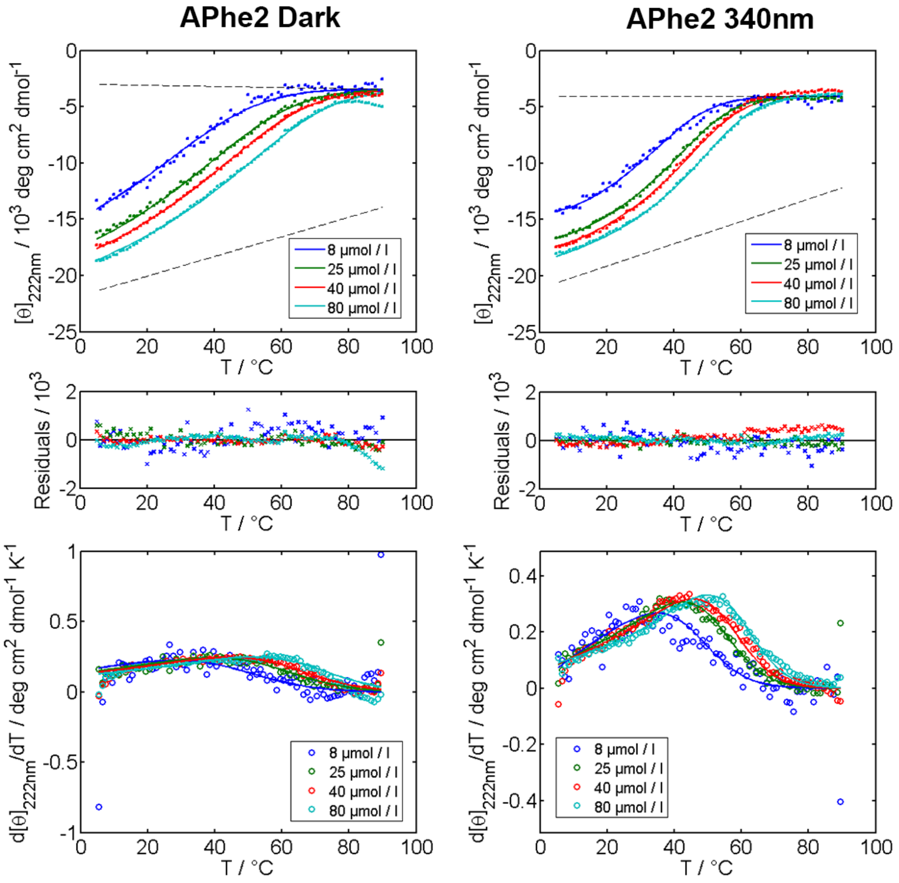

Figure S3: Fitting of CD melting curves (top) for peptide **K_3-APhe2** with E_3GY in the dark adapted (left) and 340 nm irradiated (right) states performed by the FitDis! software program. Residuals (middle) and derivatives (bottom) of the best fitting (dimeric) model are shown. Measurements were performed in PBS.

Table S1: Fit results of thermal unfolding curves for K_3 peptides containing azophenylalanine amino acids with binding partner E_3GY, in either the dark adapted or 340 nm irradiated state.

**Coiled-coil system**	**K_3-APhe1** Dark	**K_3-APhe1** 340 nm	**K_3-APhe2** dark	**K_3-APhe2** 340 nm
**$\Delta H^{\circ a}$ (kJ mol^{-1})**	224.7 ± 12.4	261.4 ± 7.4	175.0 ± 12.1	259.0 ± 9.7
**$T^{\circ b}$ (°C)**	123 ± 3.1	114.1 ± 1.5	142.5 ± 5.4	102.1 ± 2.1
**ΔC_p (kJ mol^{-1} k^{-1})**	1.69 ± 0.22	2.46 ± 0.15	1.07 ± 0.17	2.65 ±0.22
**θ_F (deg cm^2 dmol^{-1})**	-22,067 ± 413	-23,009 ± 358	-21,790 ± 548	-21,112 ± 447
**m_F**	61.3 ± 16.7	59.9 ± 14.03	87.2 ± 27.1	99.1 ± 20.6
**θ_u (deg cm^2 dmol^{-1})**	-3,566 ± 1062	-2,830 ± 876	-2,986 ± 887	-4,078 ± 545
**m_u**	-7.4 ± 13.0	-15.0 ± 10.7	-5.0 ± 11.0	0.0 ± 7.0
**ΔG_{20}^{c} (kJ mol^{-1})**	33.5	32.8	29.97	30.94
**ΔS_{20}^{c} (J mol^{-1} k^{-1})**	57	-8.95	46.31	36.7
**ΔH_{20}^{c} (kJ mol^{-1})**	50.23	30.21	43.54	41.70
**K_f^{c} (M^{-1}, 20 °C)**	9.19 10^5	7.09 10^5	2.18 10^5	3.26 10^5
**K_u^{c} (µM, 20 °C)**	**1.09**	**1.41**	**4.58**	**3.07**
**Dark/340nm PSS**		1.30		0.67
**Adjusted R^2**	0.997	0.998	0.996	0.997
**RMSE**	300.1	248.2	298.9	248.5

[a] $\Delta H°$ and T° are the enthalpy and temperature where $\Delta G = 0$ and $K_U = K_F = 1$. [b] ΔC_P is the change in heat capacity upon unfolding. [c] Binding model at 20 (°C).

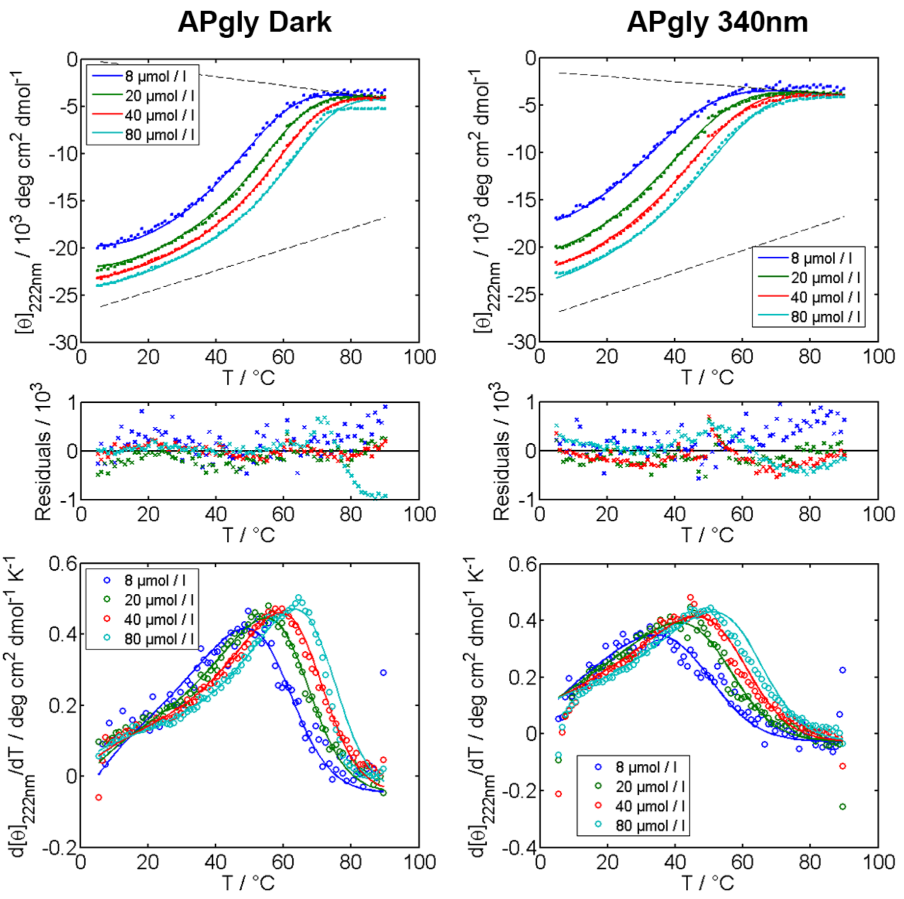

Figure S4: Fitting of CD melting curves (top) for peptide **K_3-APgly** with E_3GY in the dark adapted (left) and 340 nm irradiated (right) states performed by the FitDis! software program. Residuals (middle) and derivatives (bottom) of the best (dimeric) fitting model are shown. Measurements were performed in PBS.

Table S2: Fit results of thermal unfolding curves for K_3 peptides containing azophenylglcyine with coiled-coil binding partner E_3GY, in either the dark adapted or 340 nm irradiated state

**Coiled-coil system**	**K_3-APgly** dark	**K_3-APgly** 340 nm
**$\Delta H^{\circ a}$ (kJ mol^{-1})**	285.2 ± 6.5	236 ± 7.1
**$T^{\circ b}$ (°C)**	113 ± 1.2	110.5 ± 1.9
**ΔC_p (kJ mol^{-1} k^{-1})**	2.8 ± 0.13	2.15 ± 0.14
**θ_F (deg cm^2 dmol^{-1})**	-26877 ± 353	-27513 ± 520
**m_F**	112 ± 12.6	119 ± 23.9
**θ_u (deg cm^2 dmol^{-1})**	0 ± 1153	-1424 ± 687
**m_u**	-46.6 ± 13.9	-28 ± 8.7
**$\Delta G_{20}{}^{c}$ (kJ mol^{-1})**	34.5	30.7
**$\Delta S_{20}{}^{c}$ (J mol^{-1} k^{-1})**	-33	37.9
**$\Delta H_{20}{}^{c}$ (kJ mol^{-1})**	24.8	41.8
**$K_f{}^{c}$ (M^{-1}, 20 °C)**	1.38 10^6	2.97 10^5
**$K_u{}^{c}$ (µM, 20 °C)**	**0.72**	**3.37**
**Dark/340nm PSS**		4.65
**Adjusted R^2**	0.998	0.998
**RMSE**	274.3	288.7

[a] $\Delta H°$ and T° are the enthalpy and temperature where $\Delta G = 0$ and $K_U = K_F = 1$. [b] ΔC_P is the change in heat capacity upon unfolding. [c] Binding model at 20 (°C).

Chromatogrpahy traces of Fmoc-APgly

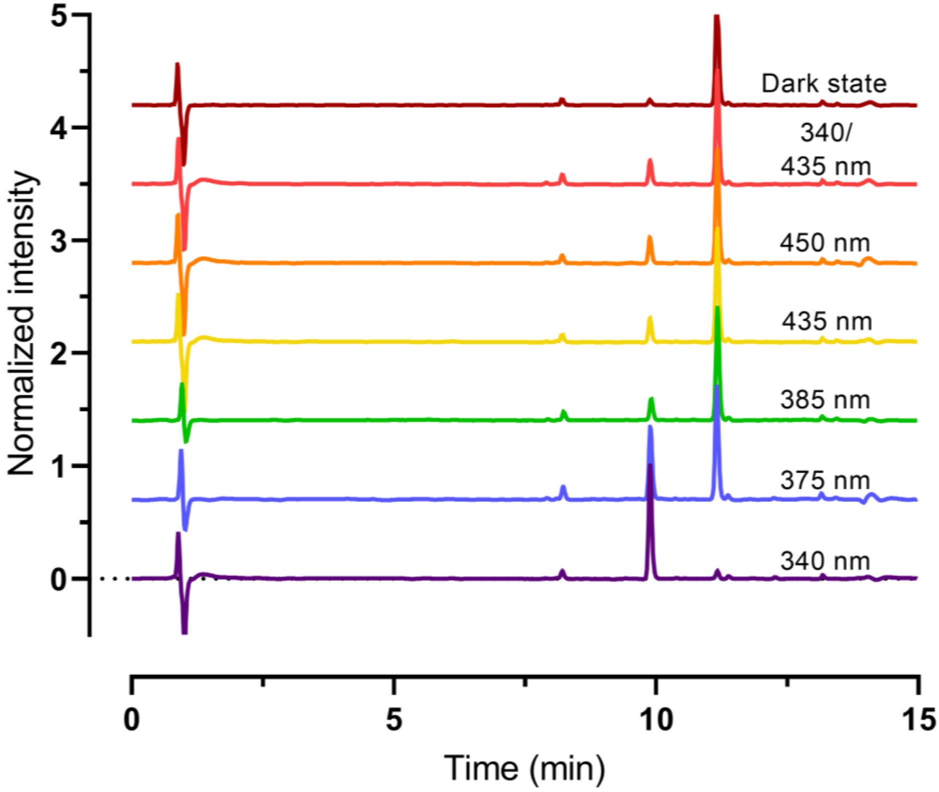

Figure S5: Chromatography traces of Fmoc-APgly in the dark state and after irradiation with different wavelengths of light. Illumination studies of the amino acid were performed in MeCN at 20 °C.

Amino acid force field parametrization

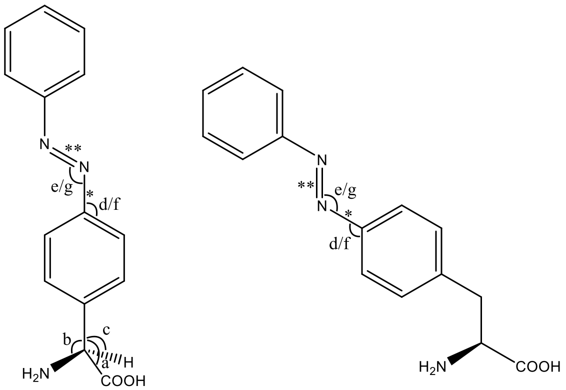

Table S3: Bond lengths, angles and dihedral angles for new atom types required for the integration of **APgly** (top left) and **APhe1** (top right) into the AMBER-96 force field for molecular dynamics simulations of azobenzene-amino acids. Bond lengths and angles as computed at BLYP-D3(BJ)/TZ2P.

	**Bond type**	**Residue**	**Bond length (nm)**	**Force constant (kJ mol^{-1} nm^{-1})**	**Adapted from**
*	CA-NX	All	0.14398	357313.6	CA-NA
**	NX-NX	All	0.14354	357313.6	CA-NA

	**Angle type**	**Residue**	**Angle (degrees)**	**Force constant (kJ mol^{-1} rad^{-1})**	**Adapted from**
a	CA-CT-C	Diazophenylglycine	109.139	527.184	CA-CT-CT
b	CA-CT-N	Diazophenylglycine	110.762	527.184	C-CT-N
c	CA-CT-H1	Diazophenylglycine	108.439	418.400	CA-CT-HC
d	CA-CA-NX	Cis	119.225	585.760	NA-CN-NC
e	CA-NX-NX	Cis	121.626	585.760	NA-CN-NC
f	CA-CA-NX	Trans	121.615	585.760	NA-CN-NC
g	CA-NX-NX	Trans	115.088	585.760	NA-CN-NC

	**Dihedral type**	**Residue**	**Dihedral angle (degrees)**	**Force constant (kJ mol^{-1} rad^{-2})**	**Adapted from**
	CA-CA-CA-NX	All	180.0 (improper)	4.60240	CA-CA-CA-NT
	CA-NX-NX-CA	Cis	186.5 (proper)	6.27600†	X-CA-NA-X
	CA-NX-NX-CA	Trans	180.1 (proper)	6.27600†	X-CA-NA-X
	CA-CA-NX-NX	Cis	227.4 (proper)	6.27600	X-CA-NA-X
	CA-CA-NX-NX	Trans	180.1 (proper)	6.27600	X-CA-NA-X

†*Set to 1000 kJ mol^{-1} rad^{-2} for production runs*

Photocycling of K₃-APgly

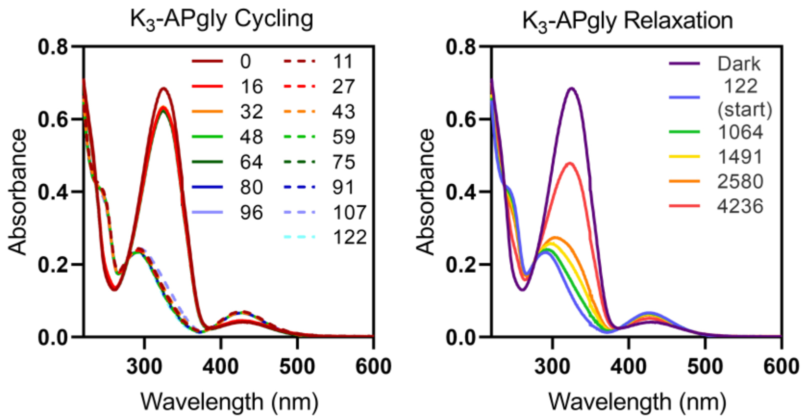

Figure S6: UV-Vis spectra of peptide **K_3-APgly** cycling between different photostationary states (left) and relaxing from the 340 nm PSS (right), which were used to prepare **Figure 4**. Spectra after illumination with 340 nm light are indicated with dotted lines (left). Graphs are numbered by time in minutes after start of the experiment. Measurements were performed at 20 °C in PBS with [peptide] = 50 µM.

MD simulations extra figures

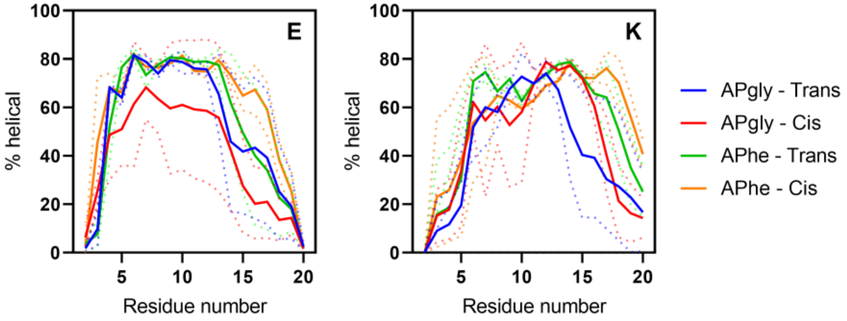

Figure S7: Percentage of helical folding (based on GROMACS' default criteria for psi/phi dihedral angles and H-bond distances) over 500 ns of simulation time, for all amino acids in peptide E (left) and peptide K (right), averaged over 3 independent MD simulations. Standard deviations are plotted as dotted lines.

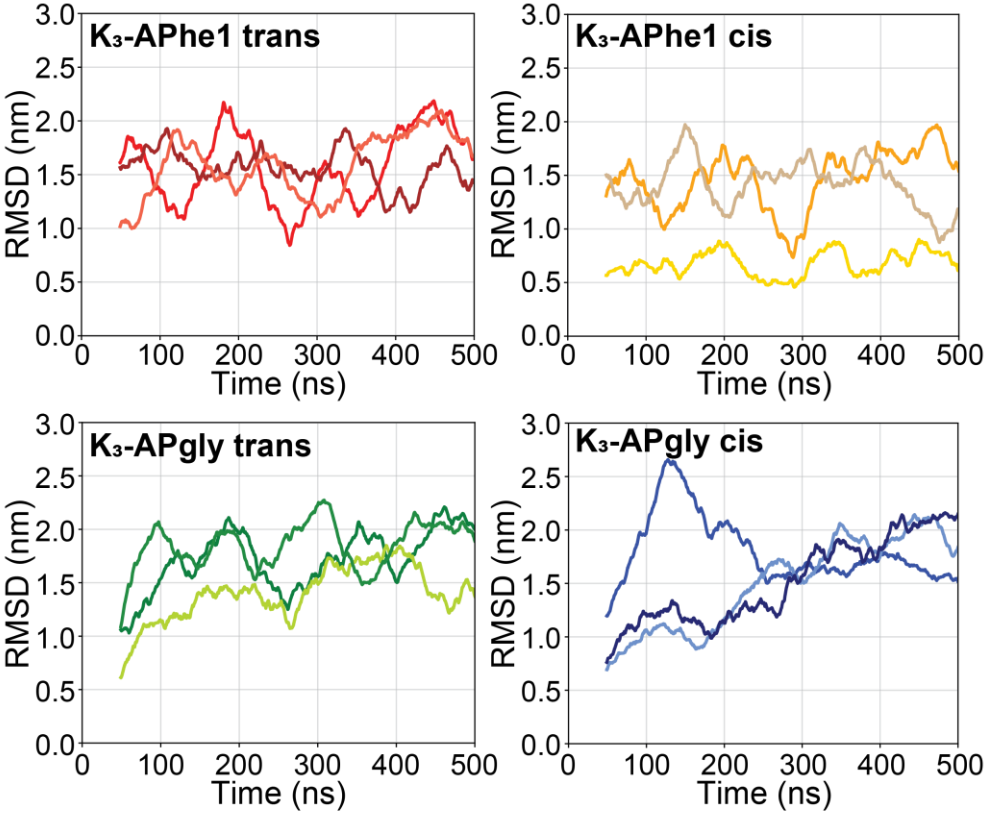

Figure S8: 50 ns running averages of the root mean square deviation (RMSD) of the coiled coil complexes during MD simulations, with respect to the initial conformation.

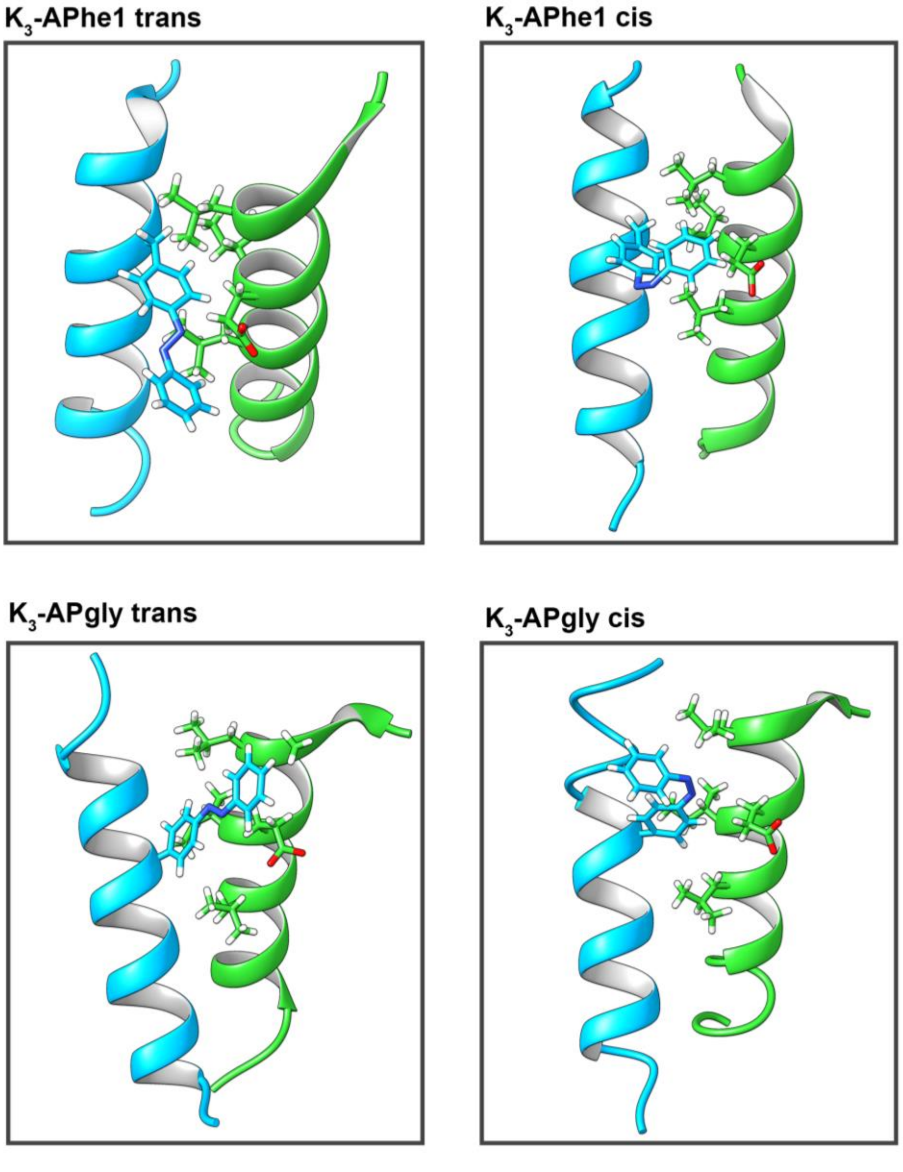

Figure S9: Snapshots from MD simulations of the coiled coil between peptide K_3 (blue) and E_3 (green), with peptide K_3 containing the photoswitchable amino acid **APhe1** or **APgly** at position 9 in the peptide sequence, in the *trans* or *cis* conformation. Peptides backbones are shown as a cartoon, with the photoswitch and all amino acid side chains of peptide E_3 within 5 Å displayed as sticks.

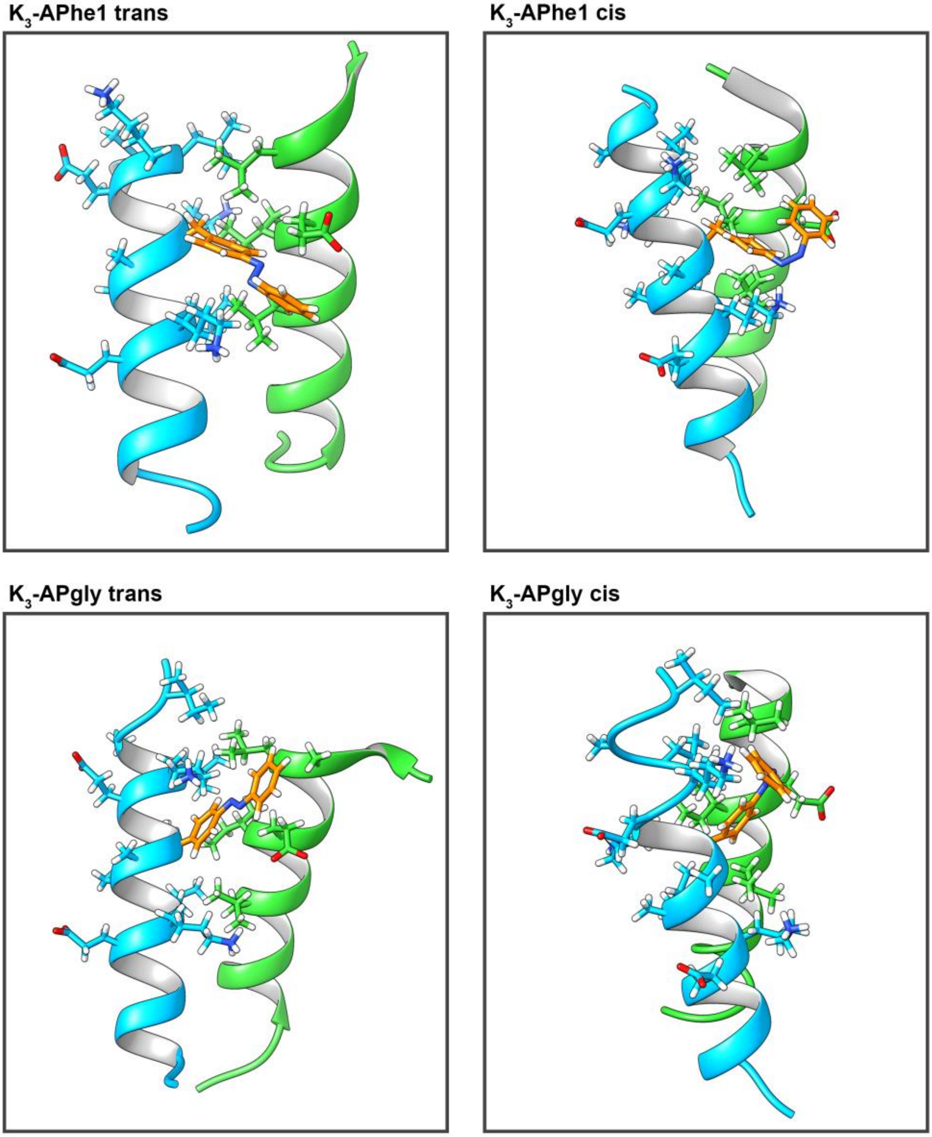

Figure S10: Snapshots from MD simulations of the coiled coil between E_3 (green) and K_3 (blue) containing **APhe1** or **APgly** at position 9 in the peptide sequence, in either the cis or *trans* conformation. Photoswitchable amino acids in peptide K_3 have been coloured orange for clarity. Peptides backbones are shown as a cartoon, with the photoswitch and all amino acid side chains within 5 Å displayed as sticks.

LC-MS Spectra of purified peptides

Table S4: Overview of the calculated masses of all peptides used in this project, and the masses found by LCMS.

Peptide name	Calculated mass (Da)	Measured mass (Da)
E_3GY	$[M + 2H^+]^{2+}$ 1272.69<br>$[M + H^+]^+$ 2544.39	1271.24<br>2545.88
K_3-P9-APhe1	$[M + 2H^+]^{2+}$ 1230.24	1228.81
K_3-P9-APhe2	$[M + 2H^+]^{2+}$ 1252.24	1250.85
K_3-P9-APgly	$[M + 2H^+]^{2+}$ 1223.23	1222.23

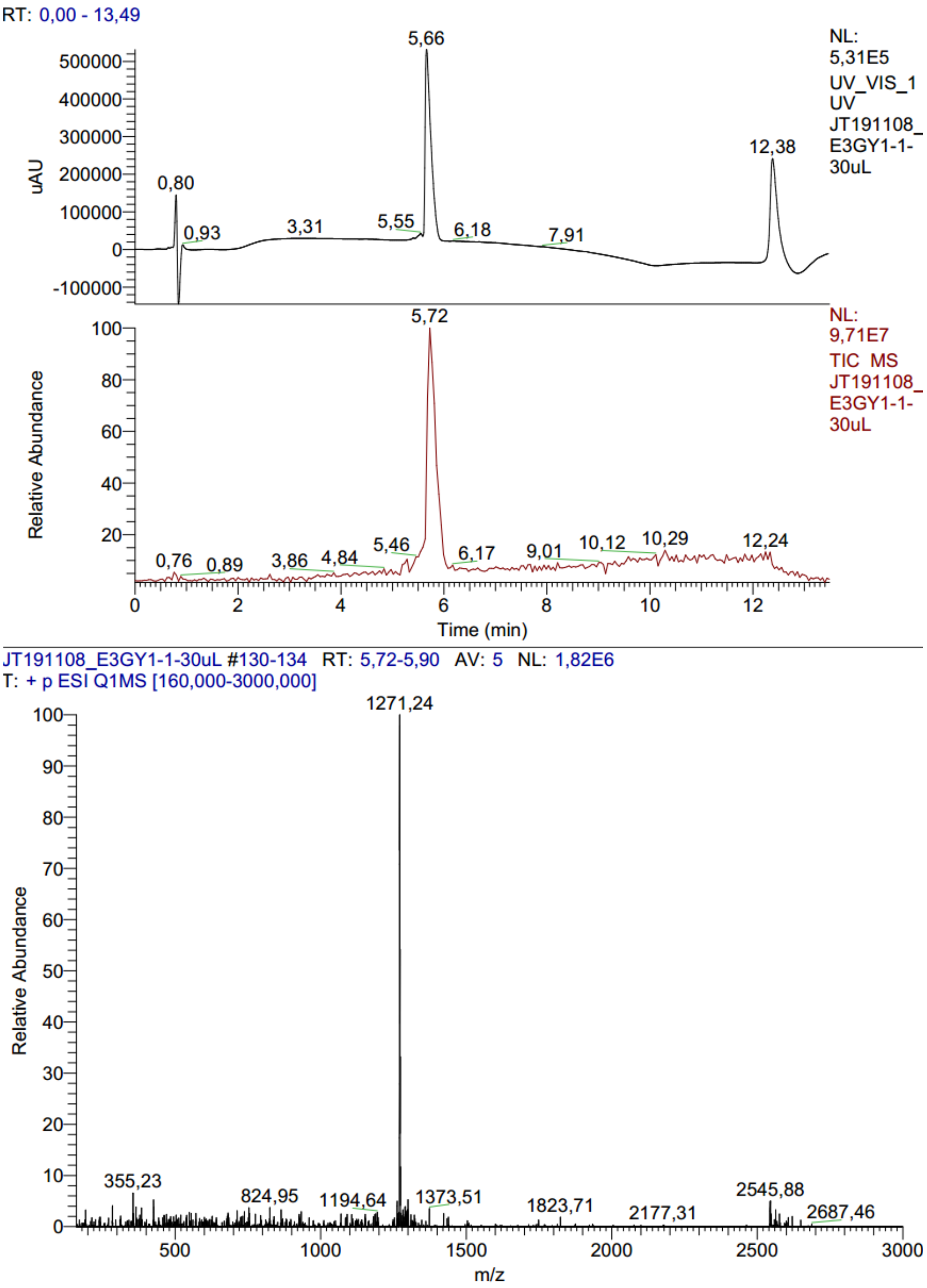

Figure S11: LC-MS spectra of pure peptide E_3GY.

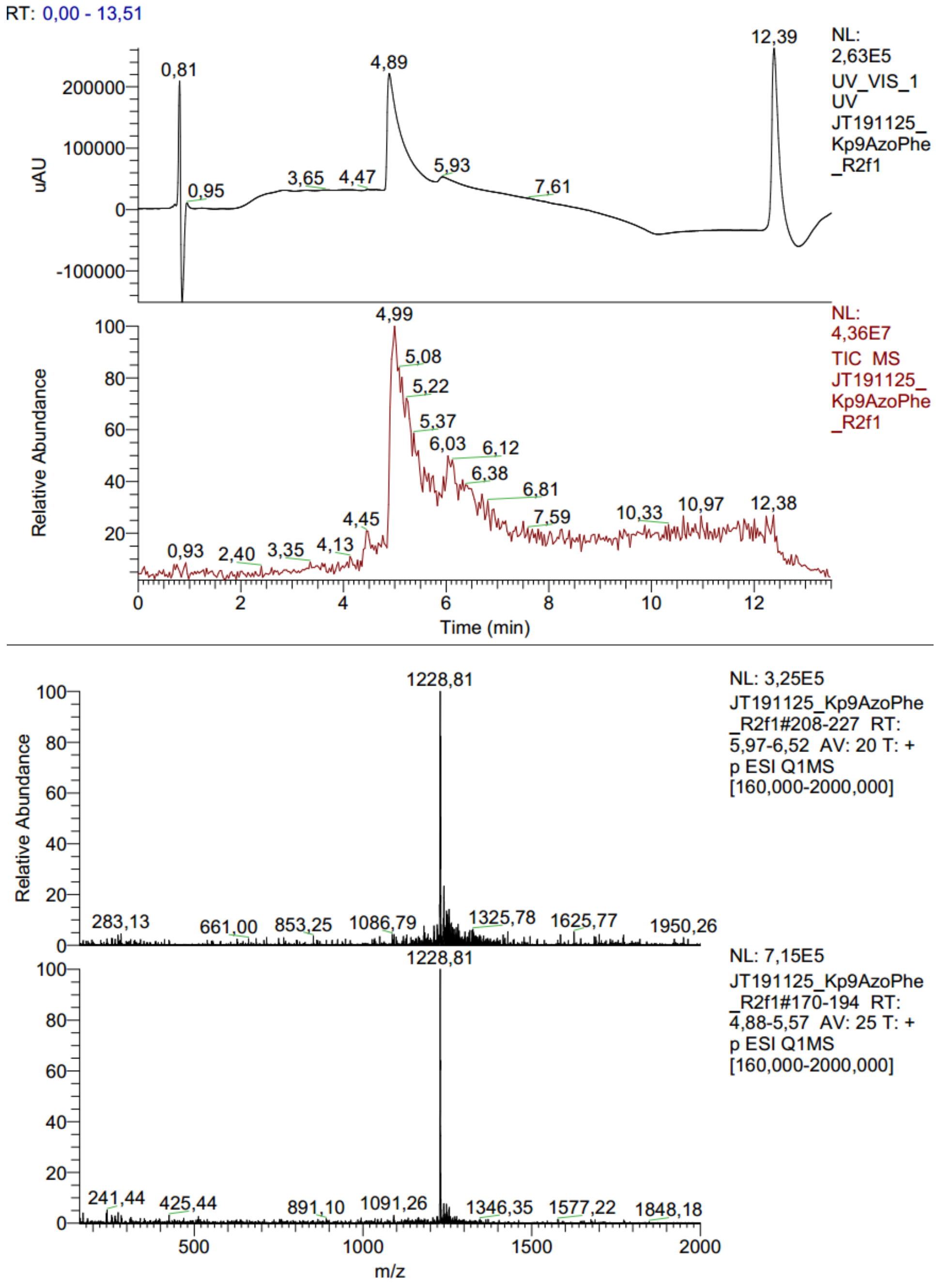

Figure S12: LC-MS spectra of pure peptide **K_3-APhe1**.

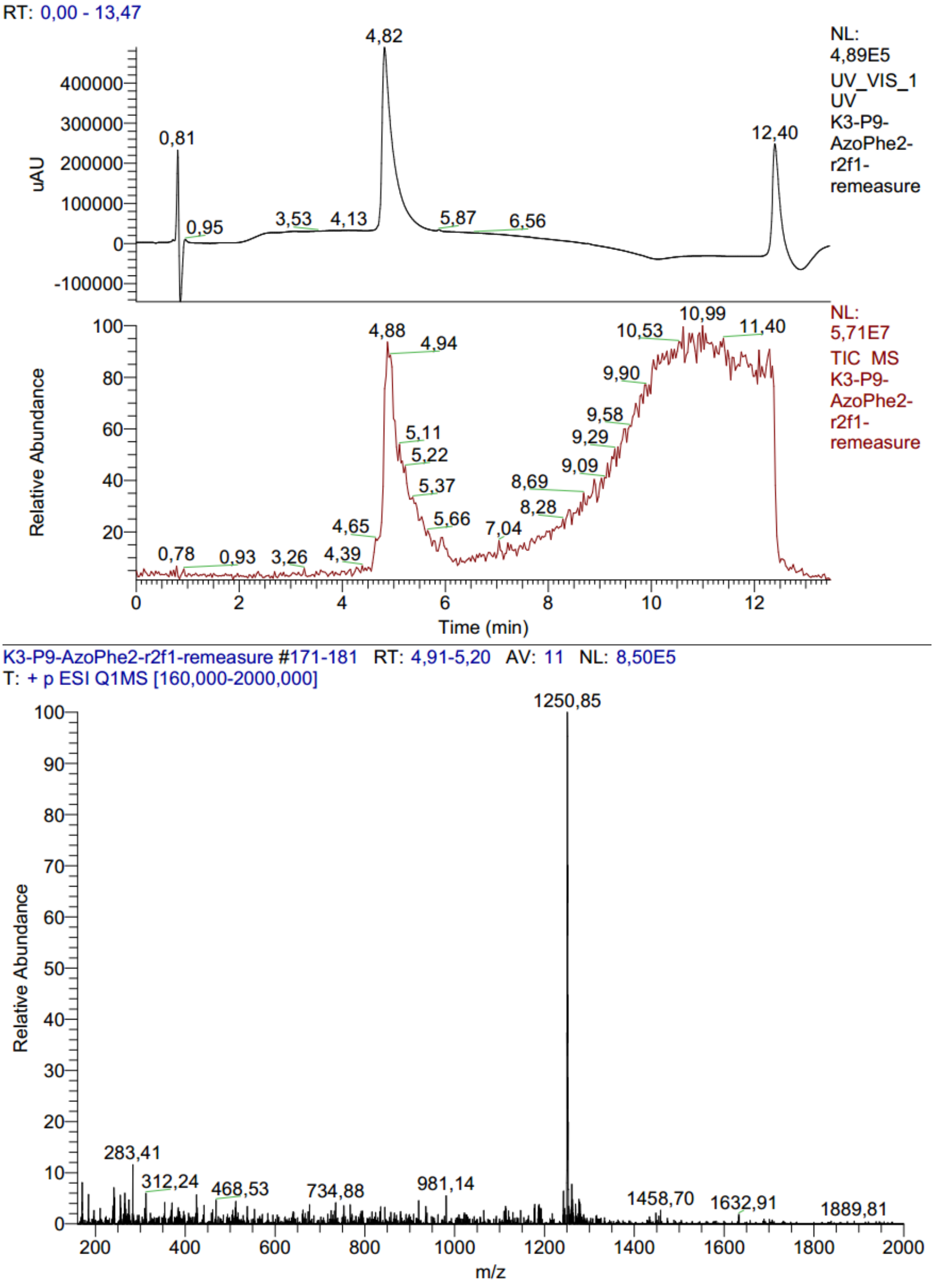

Figure S13: LC-MS spectra of pure peptide **K_3-APhe2**.

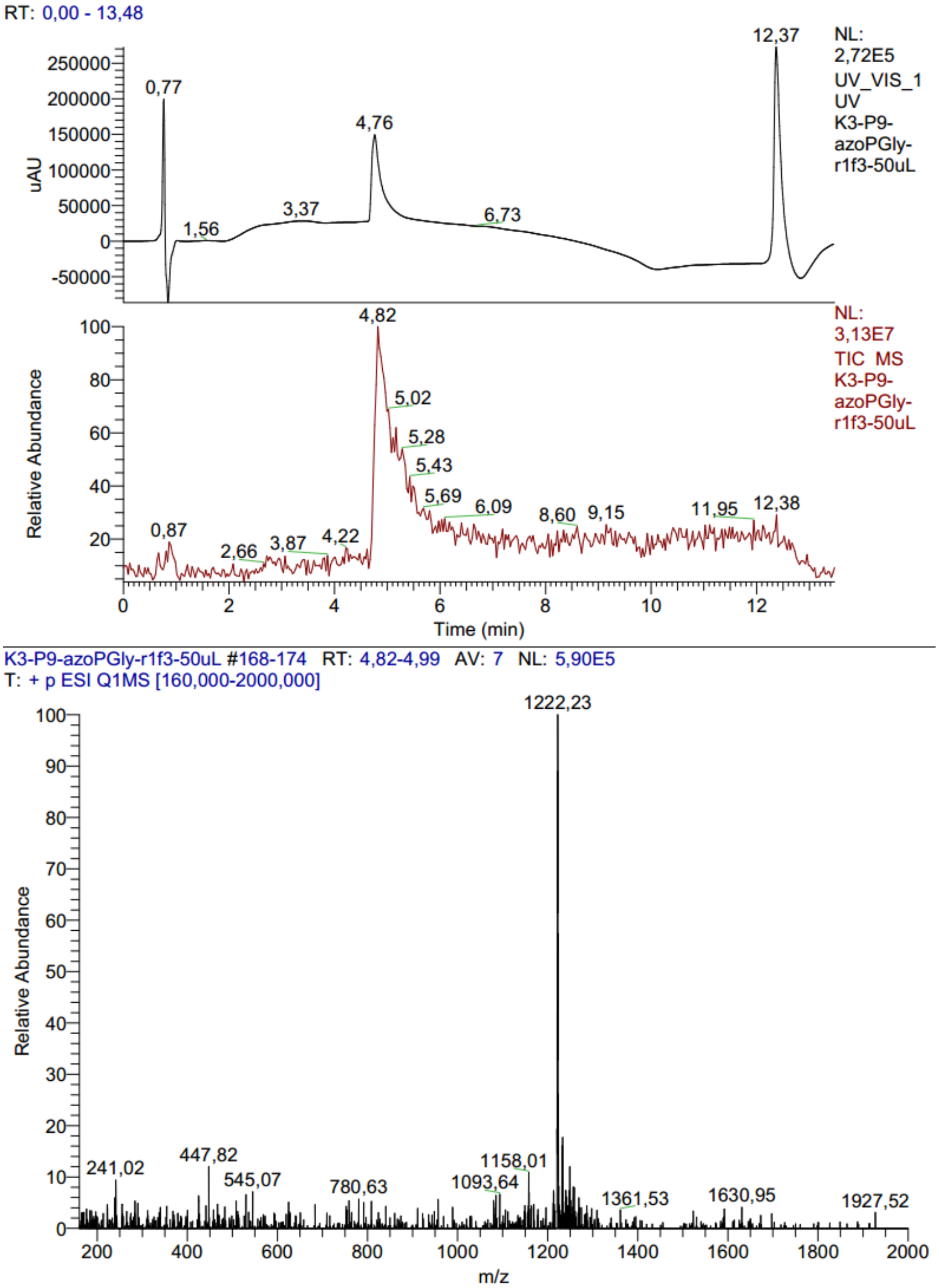

Figure S14: LC-MS spectra of pure peptide **K_3-APGly**.

LC-MS Spectra of synthetic amino acids and building blocks

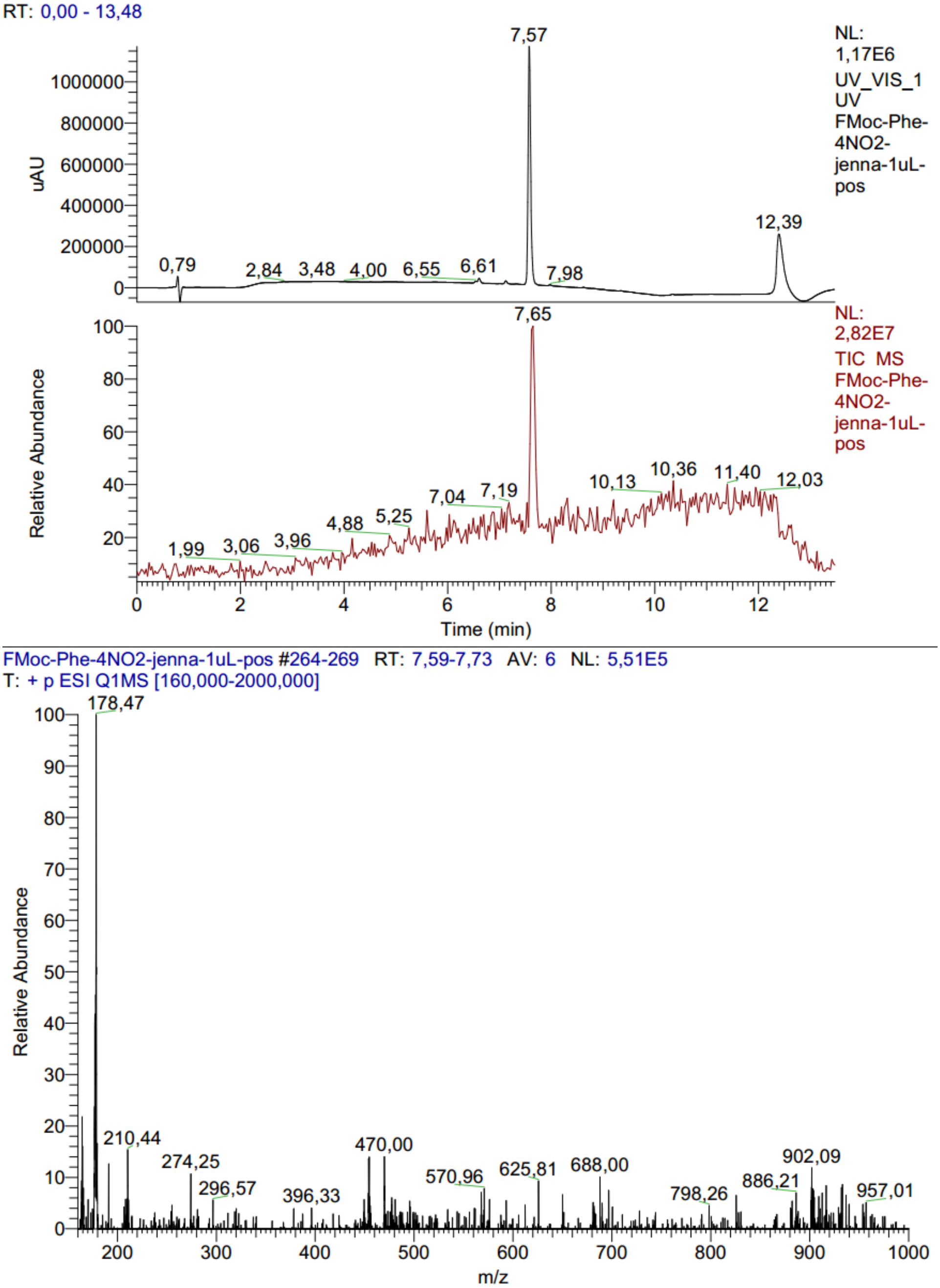

Figure S15: LC-MS spectra of pure N-fmoc-(4-nitro)-ʟ-phenylalanine (**1**).

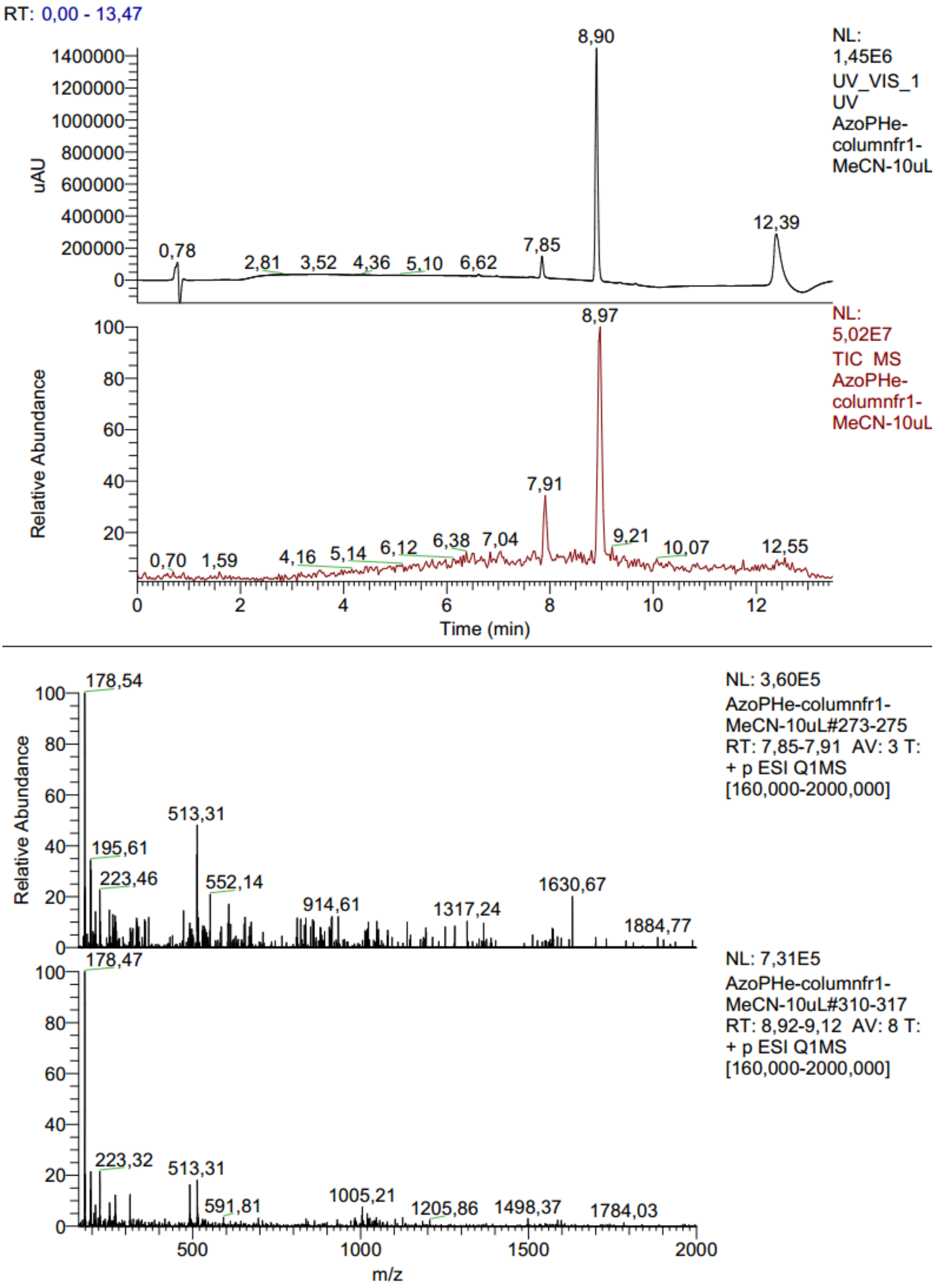

Figure S16: LC-MS spectra of pure N-fmoc-(4-phenylazo)-ʟ-phenylalanine (Fmoc-APhe1, **2**).

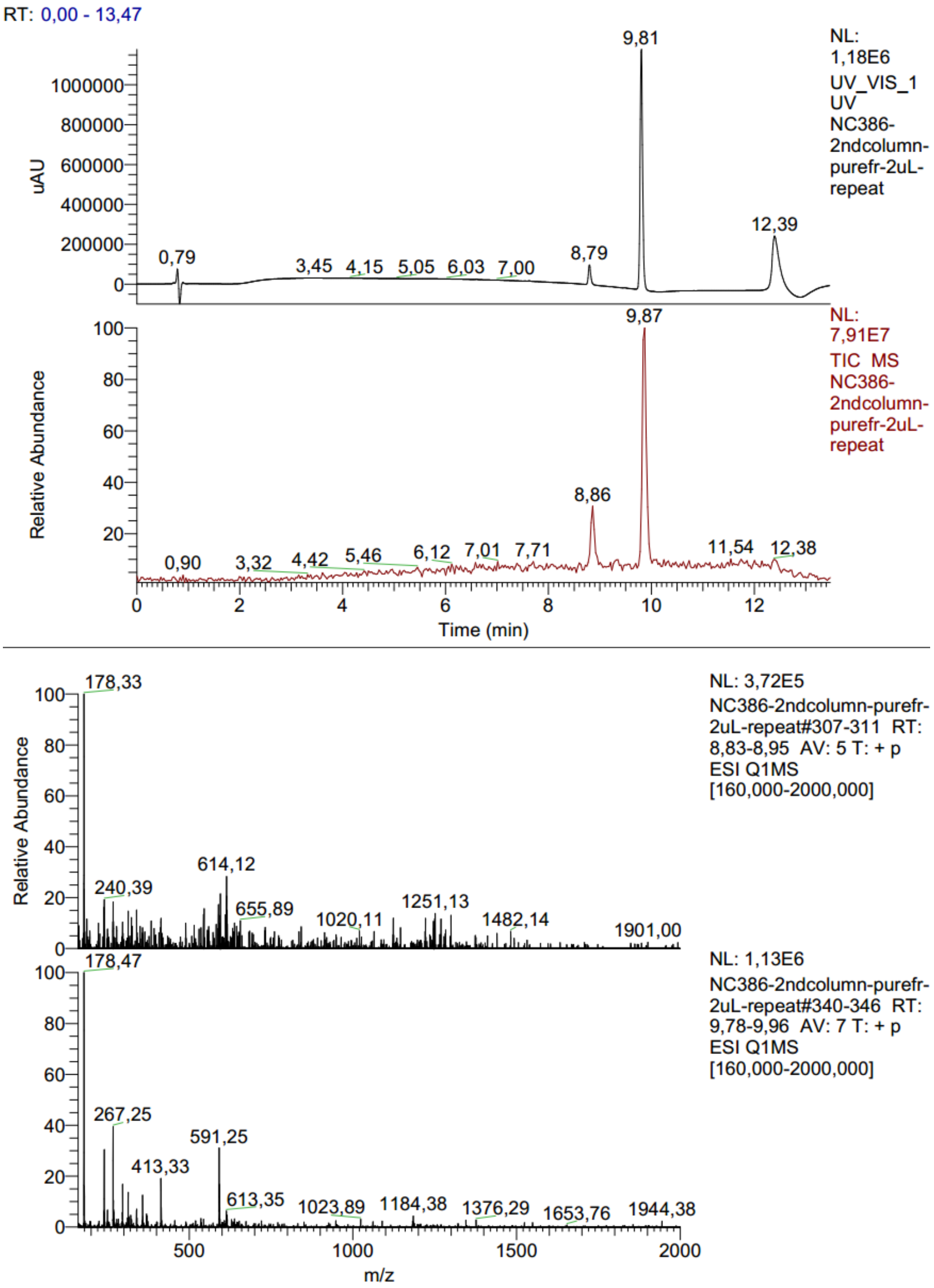

Figure S17: LC-MS spectra of pure N-fmoc-ʟ-(4-(4’-tert-Butoxycarbonyl)phenylazo)phenylalanine (Fmoc-APhe2, **3**).

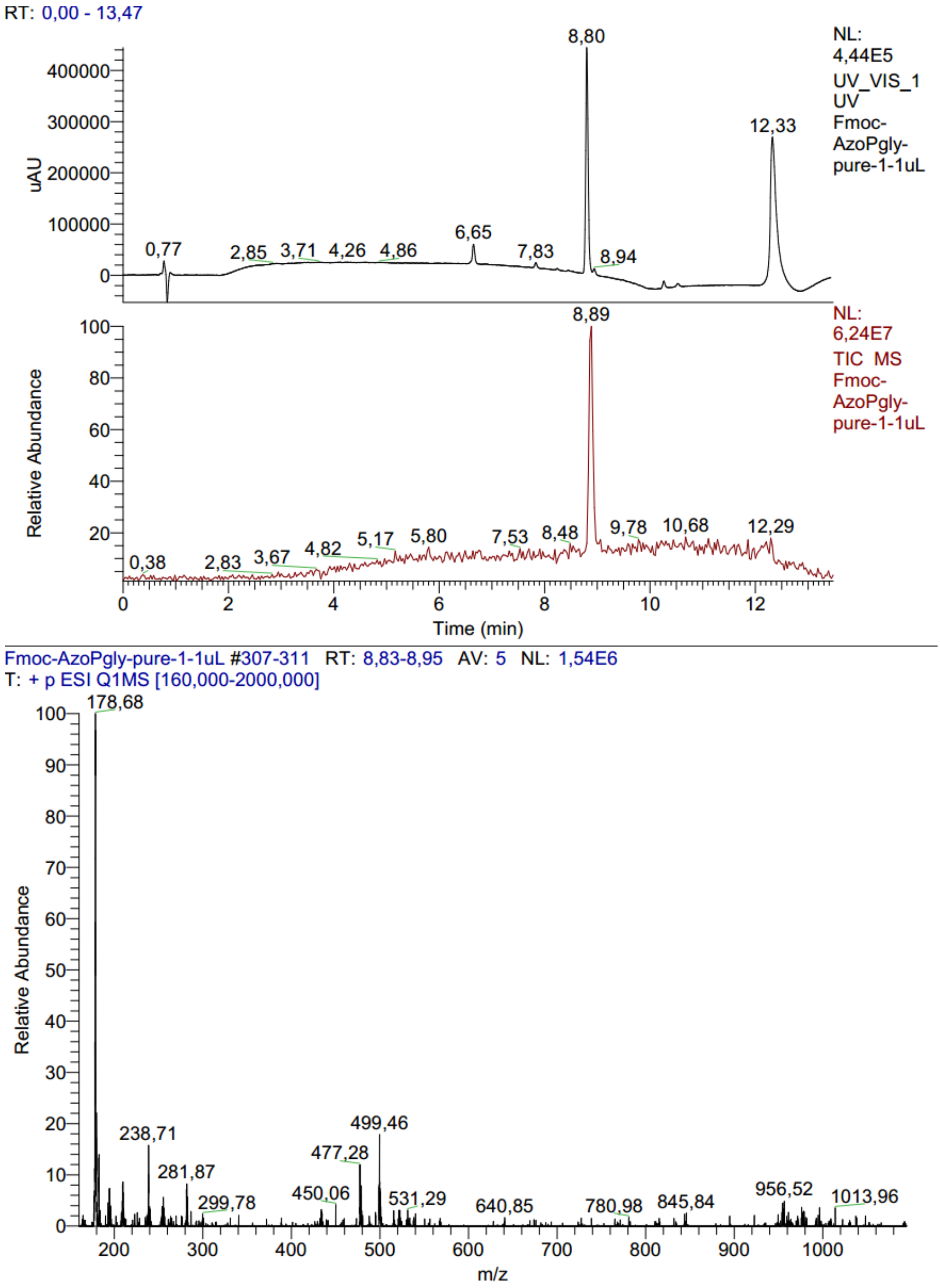

Figure S18: LC-MS spectra of pure N-fmoc-4-(phenylazo)-ʟ-phenylglycine (Fmoc-APGly, **10**).

NMR Spectra of synthetic amino acids and building blocks

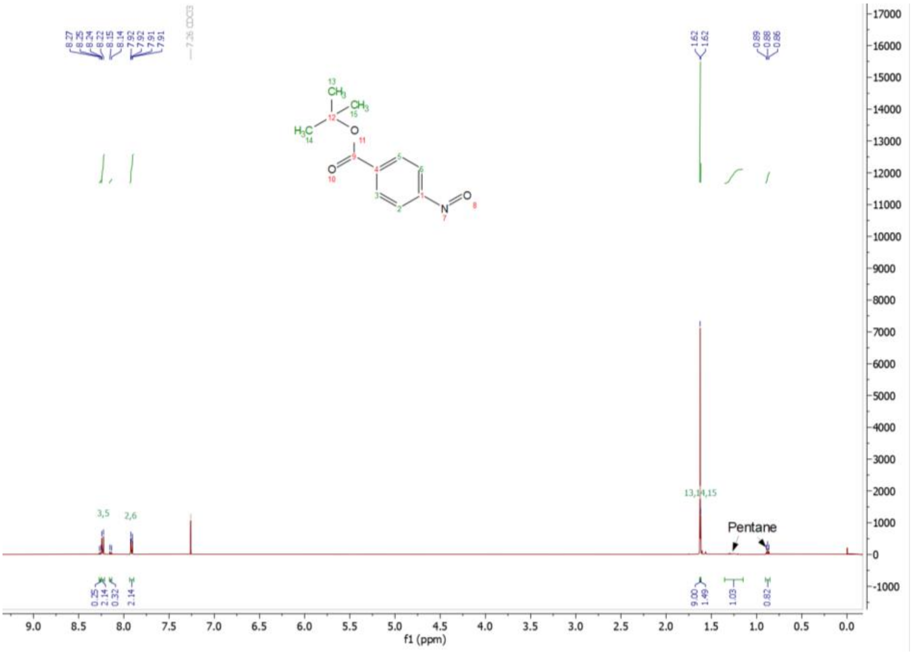

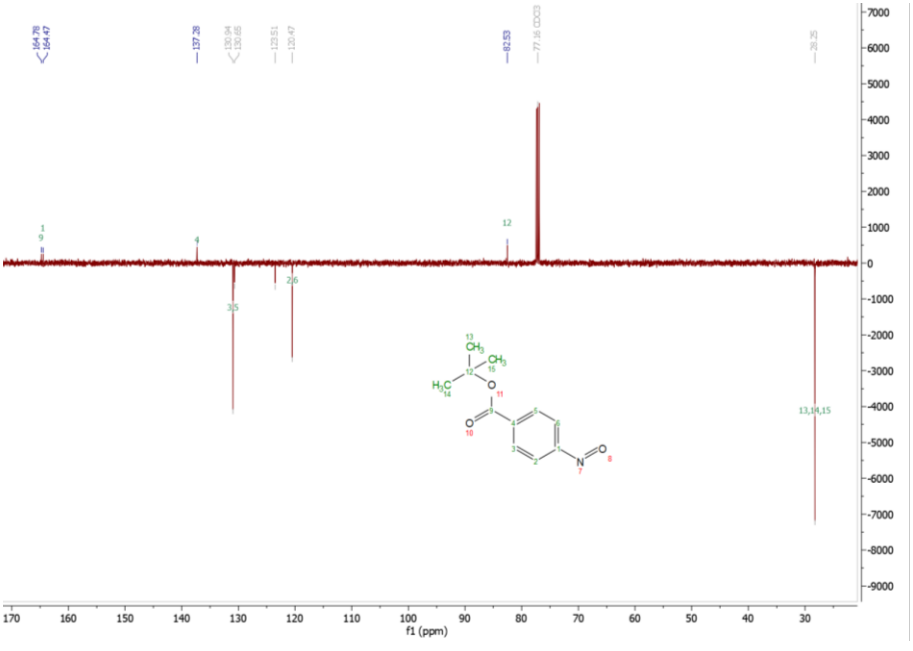

Figure S19: ^{1}H and ^{13}C NMR Spectrum of pure 4-tert-Butyl-nitrosobenzoate.

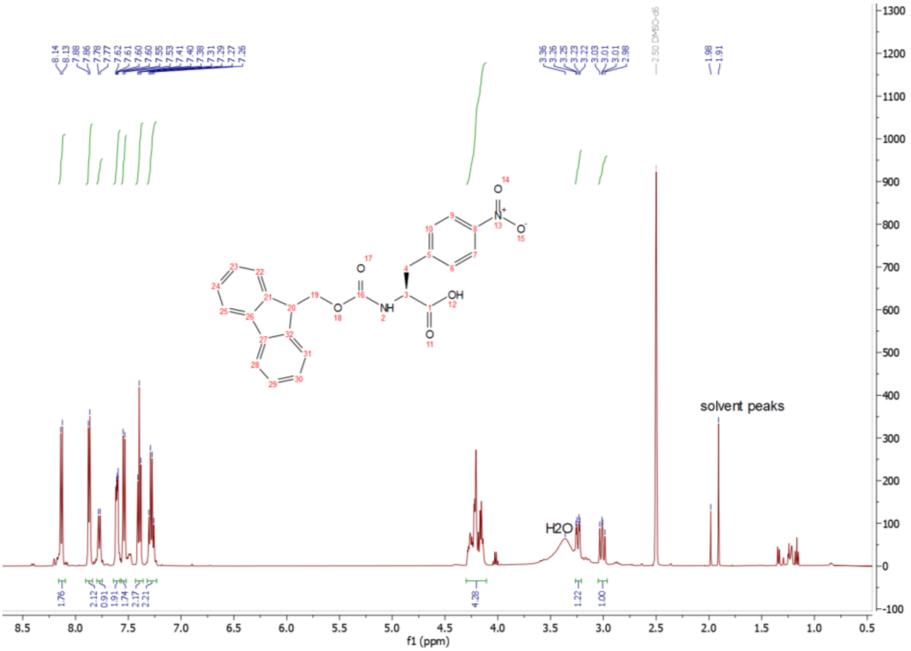

Figure S20: ^{1}H NMR Spectrum of pure N-Fmoc-4-nitro-L-phenylalanine (**1**).

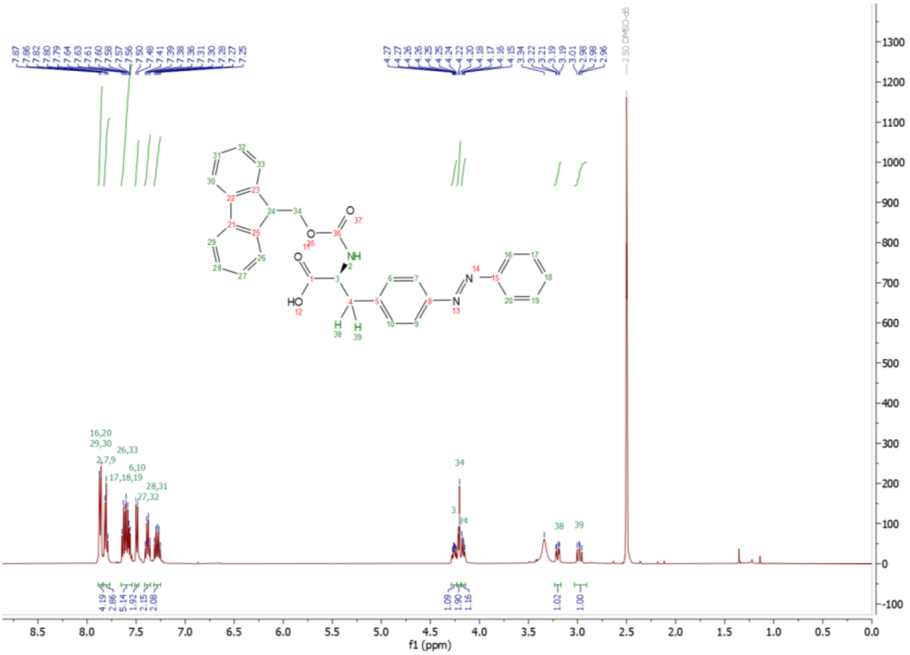

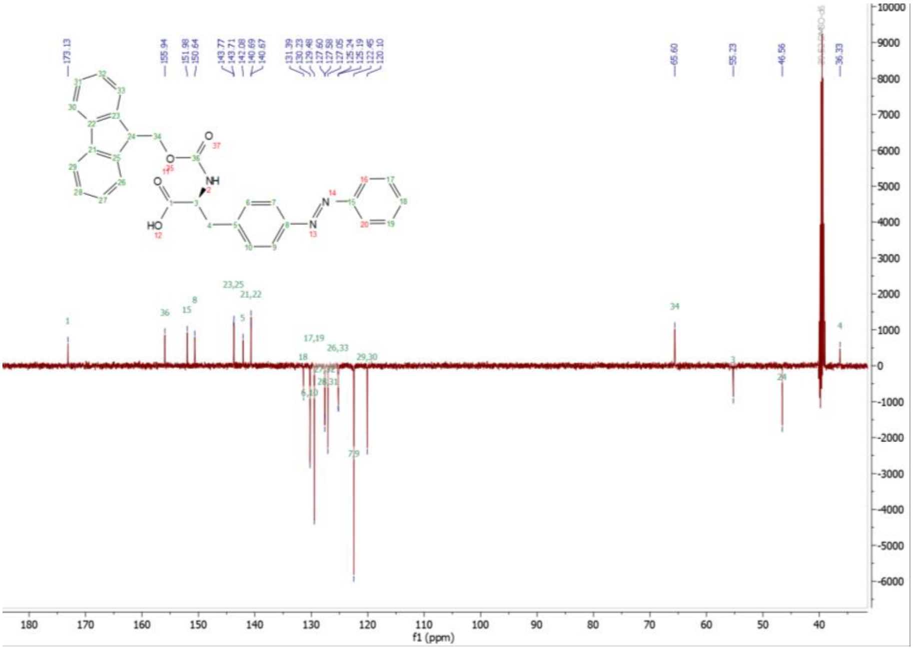

Figure S21: ^{1}H and ^{13}C NMR Spectrum of pure N-fmoc-(4-phenylazo)-L-phenylalanine, Fmoc-APhe1 (**2**).

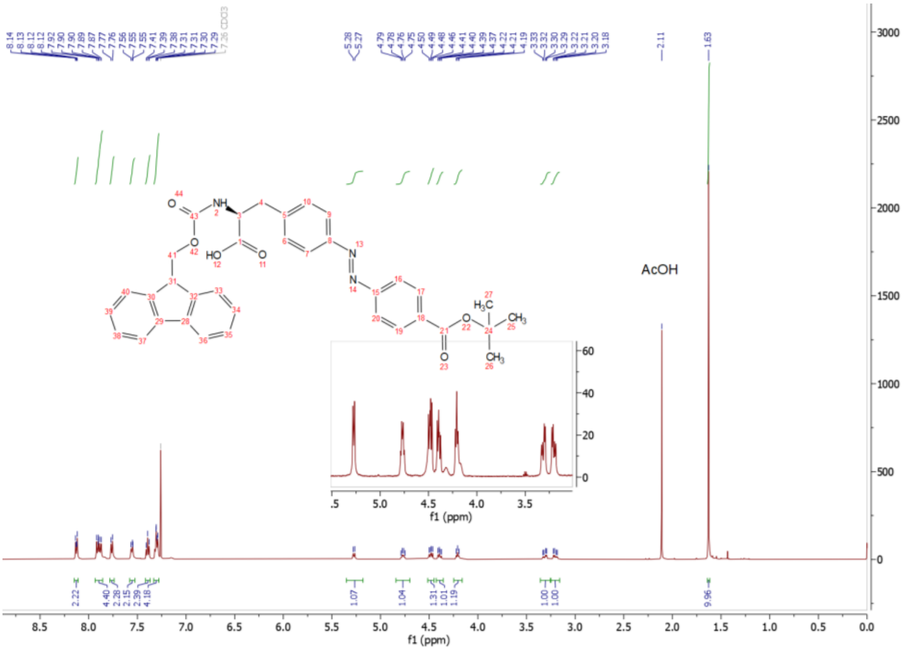

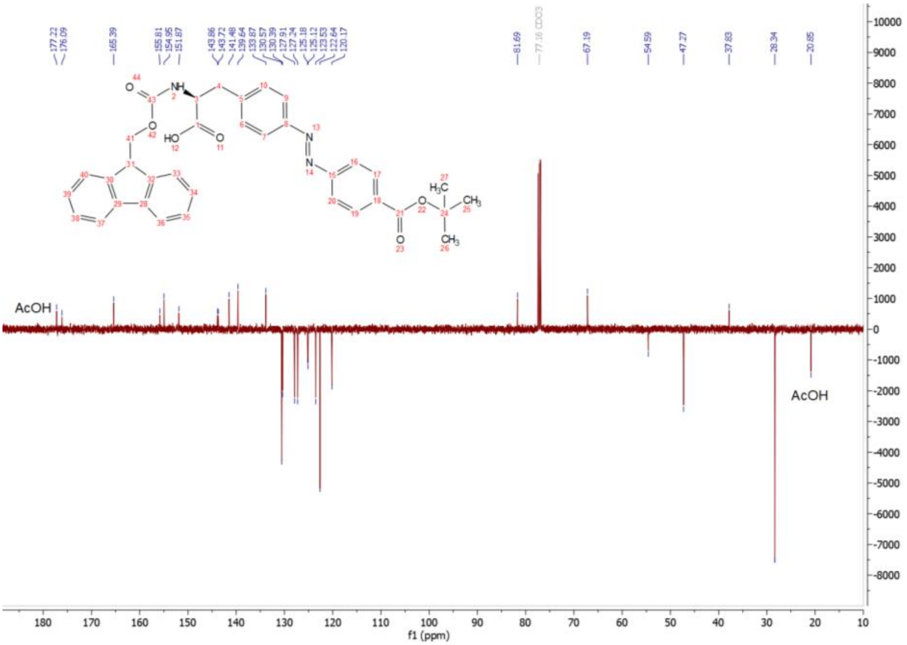

Figure S22: ^{1}H and ^{13}C NMR Spectrum of pure N-fmoc-L-(4-(4'-tert-Butoxycarbonyl)phenylazo)-phenylalanine, fmoc-APhe2 (**3**).

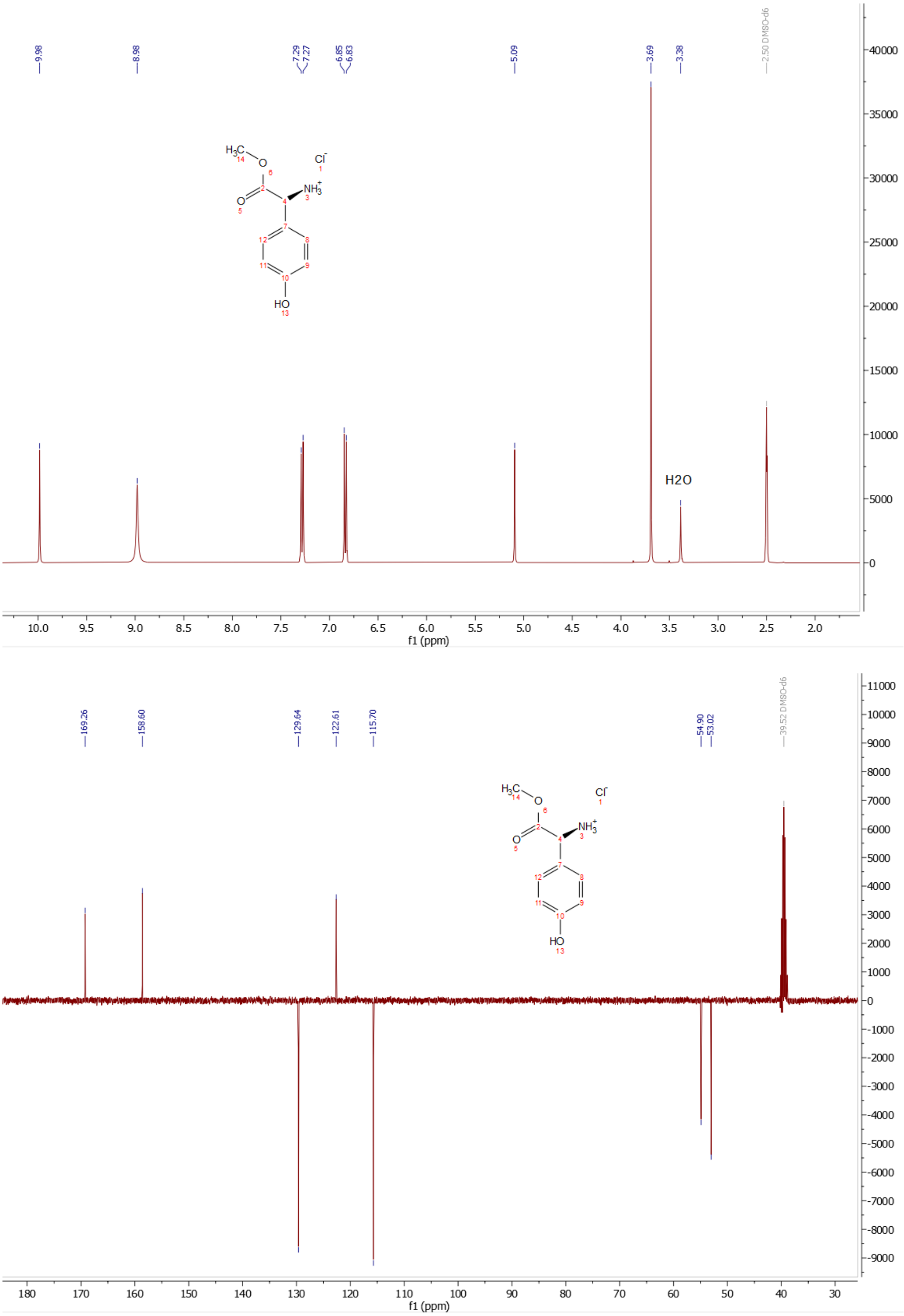

Figure S23: ^{1}H and ^{13}C NMR Spectrum of pure 4-Hydroxy-L-phenylglcyine methyl ester (**4**).

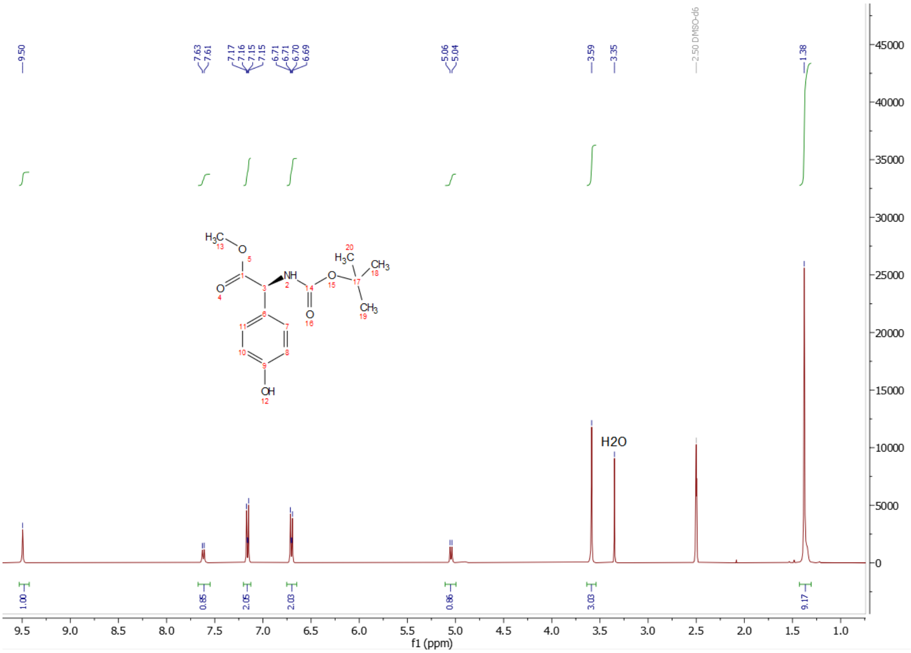

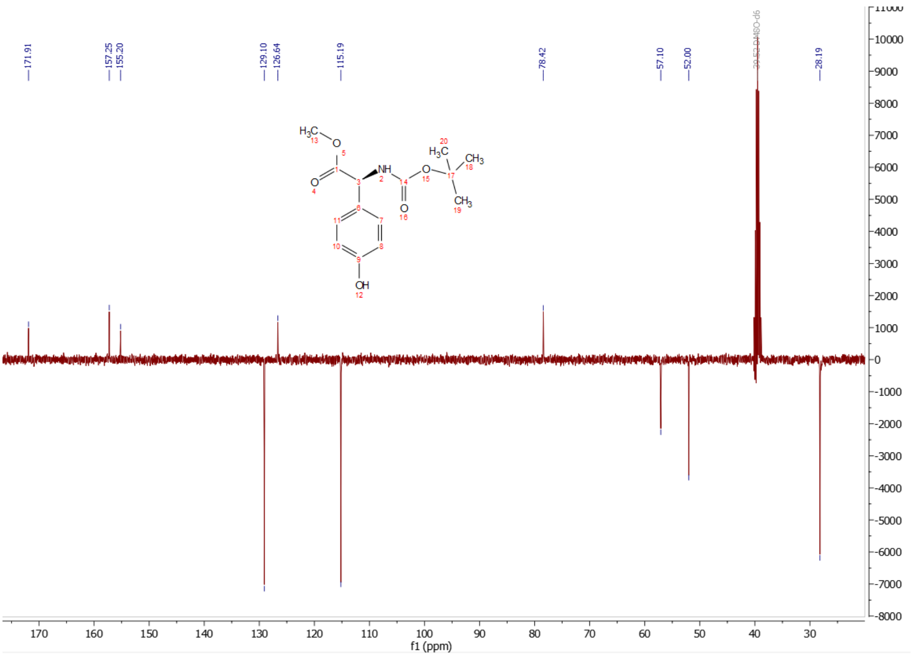

Figure S24: ^{1}H and ^{13}C NMR Spectrum of N-Boc-4-hydroxy-L-phenylglycine methyl ester (**5**).

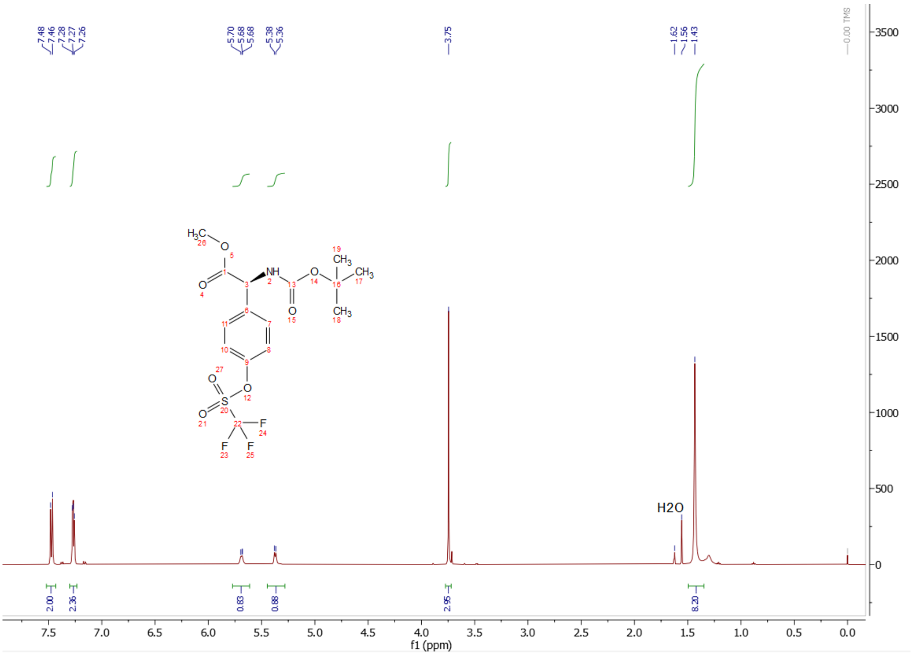
7.48
7.46
7.28
7.27
7.26
5.70
5.68
5.68
5.38
5.36
3.75
1.62
1.56
1.43
0.00 TMS
H2O
2.00
2.36
0.83
0.88
2.95
8.20
f1 (ppm)

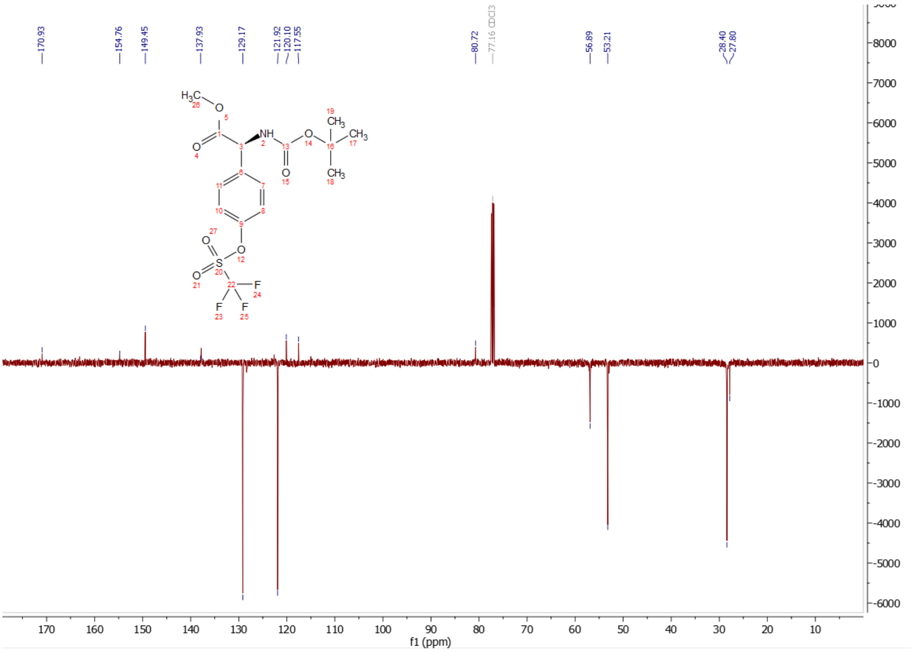
170.93
154.76
149.45
137.93
129.17
121.92
120.10
117.55
80.72
77.16 CDCl3
56.89
53.21
28.40
27.80
f1 (ppm)

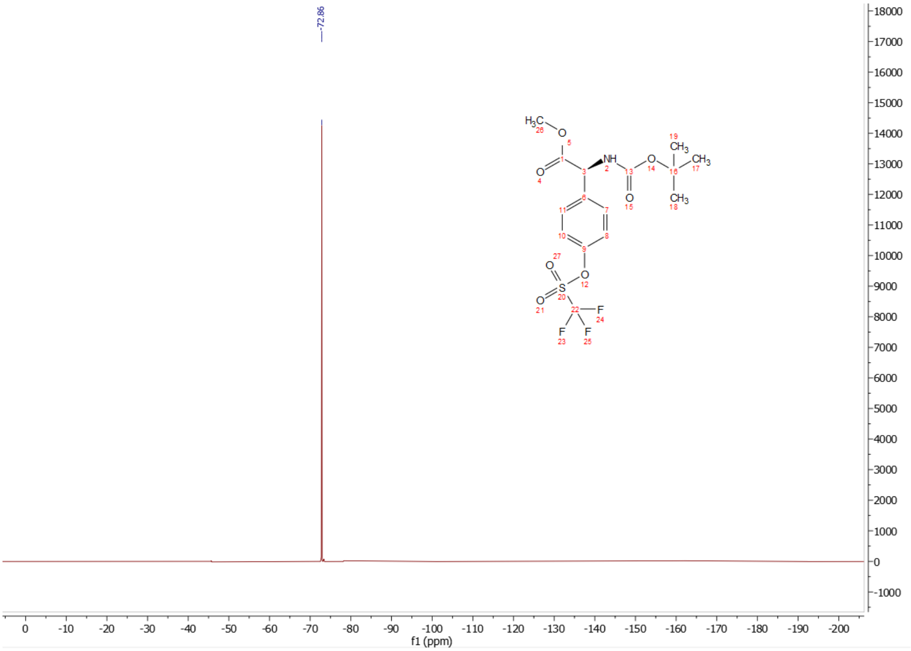

Figure S25: ^{1}H, ^{13}C and ^{19}F NMR Spectrum of N-Boc-4-(trifluoromethanesulfonate)-L-phenylglycine methyl ester (**6**).

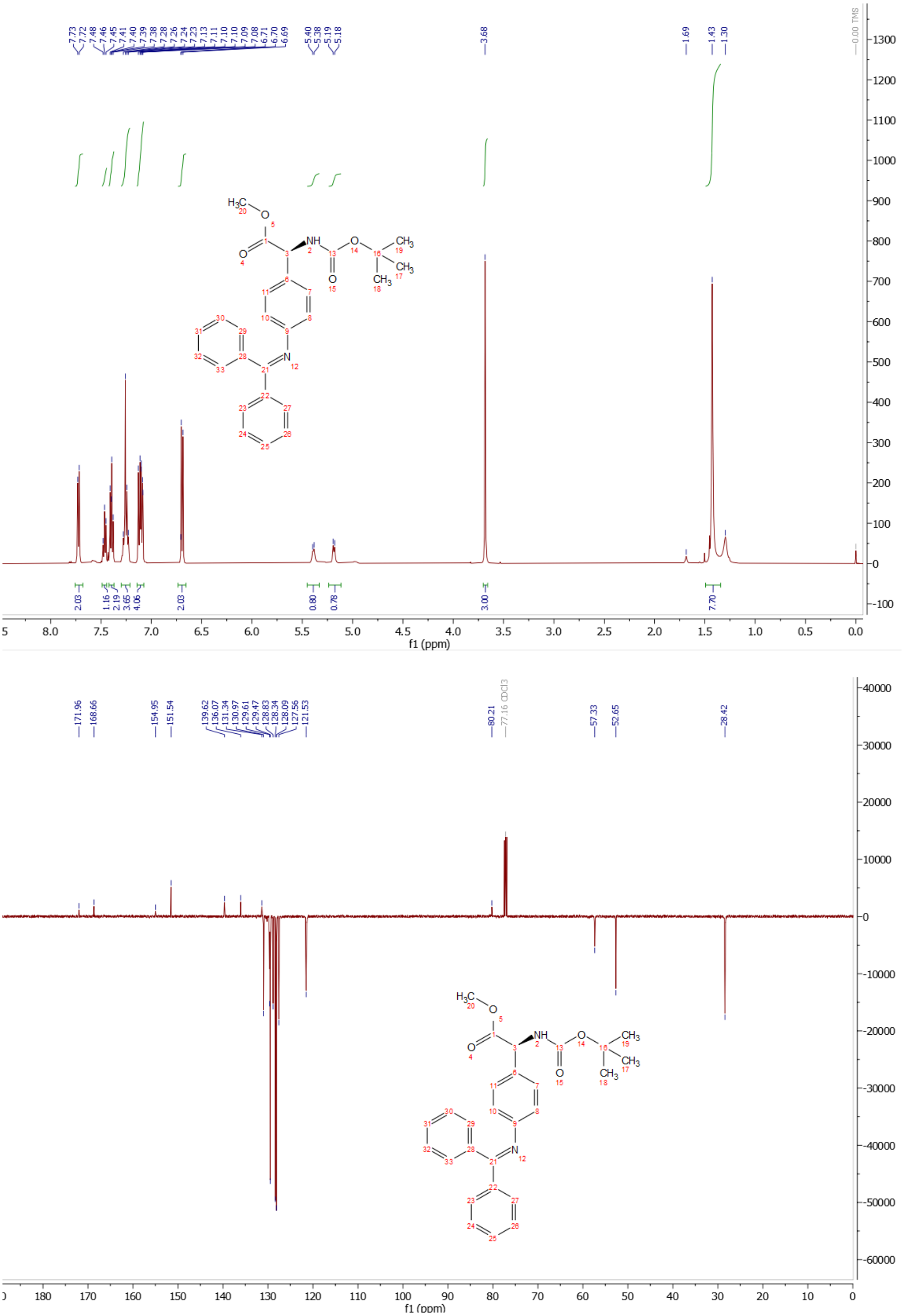

Figure S26: ^{1}H and ^{13}C NMR Spectrum of N-Boc-4-((diphenylmethylene)-amino)-ʟ-phenylglycine methyl ester (**7**).

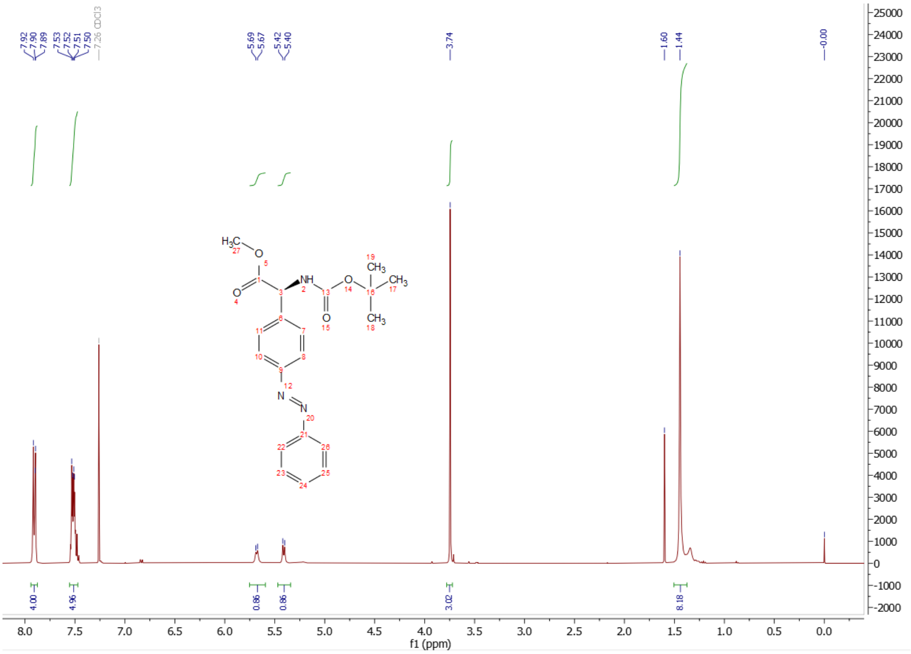

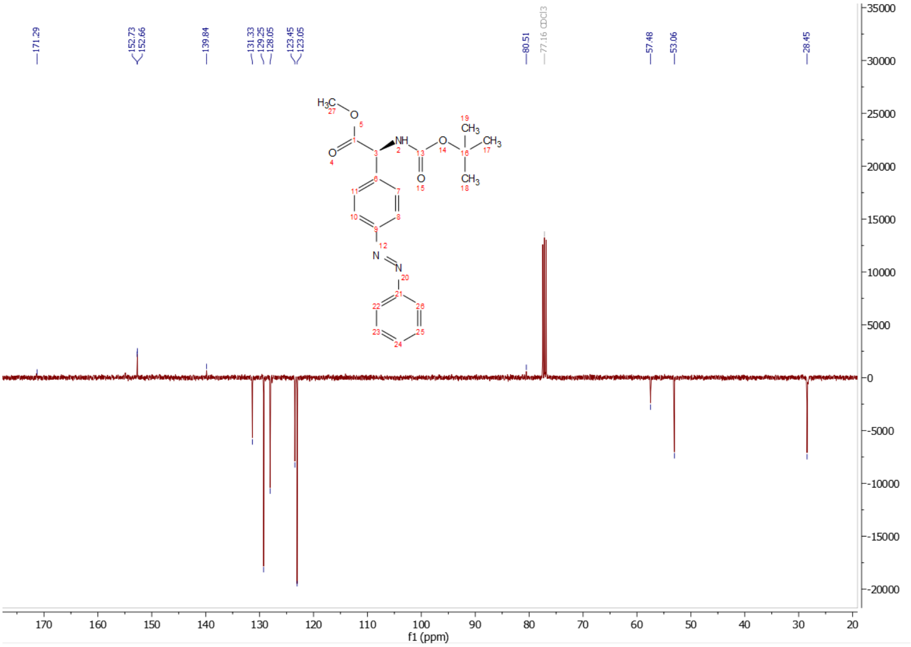

Figure S27: ^{1}H and ^{13}C NMR Spectrum of N-Boc-(4-phenylazo)-L-phenylglycine methyl ester (**9**).

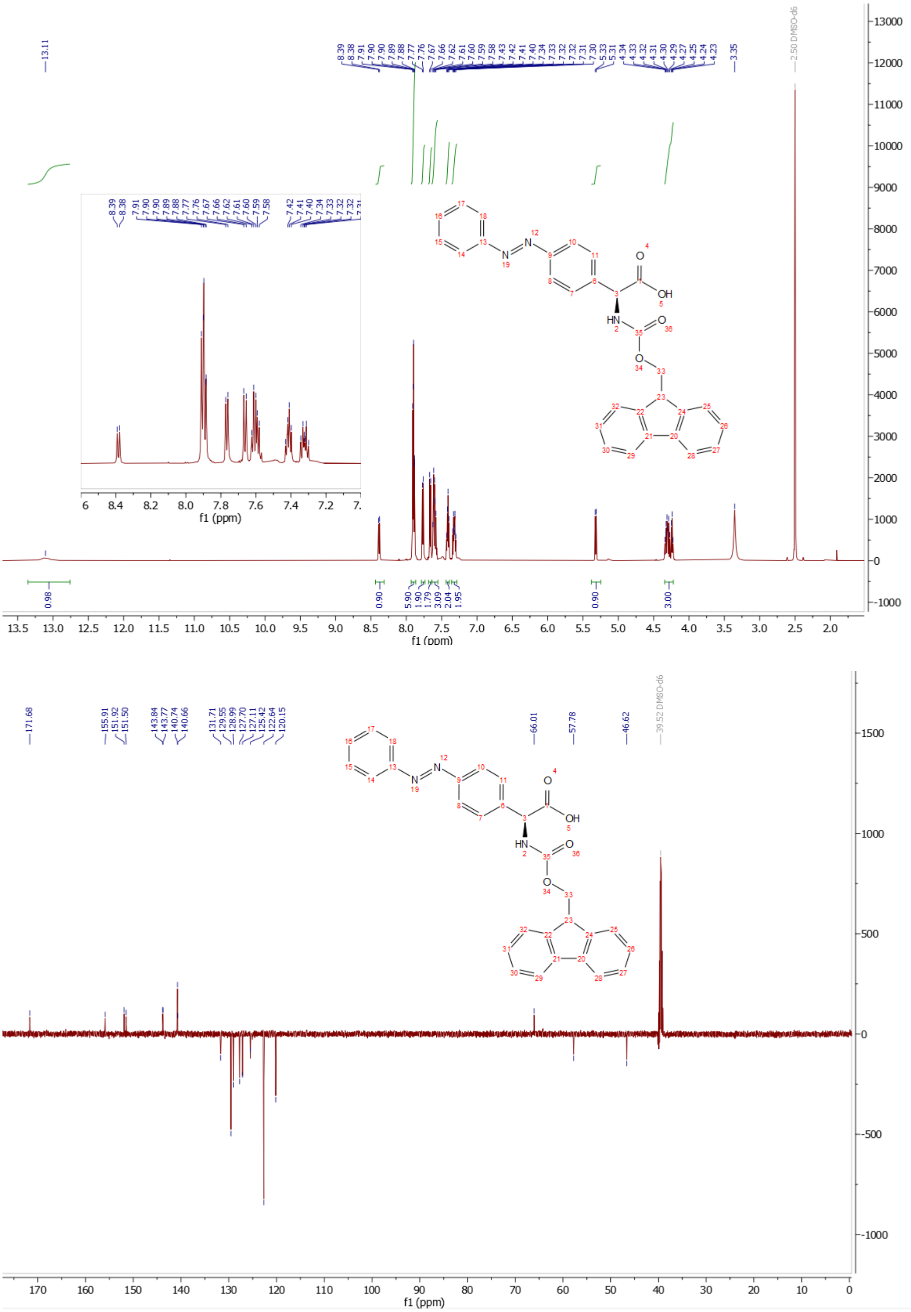

Figure S28: ^{1}H and ^{13}C NMR Spectrum of N-fmoc-4-(phenylazo)-ʟ-phenylglycine, Fmoc-APgly (**10**).
